# An improved linear prediction evolution algorithm based on topological opposition-based learning for optimization

**DOI:** 10.1016/j.mex.2023.102511

**Published:** 2023-12-04

**Authors:** A.M. Mohiuddin, Jagdish Chand Bansal

**Affiliations:** South Asian University, New Delhi, India

**Keywords:** Grey prediction evolutionary algorithm, Non-linear least square fitting, Opposition based learning, Optimization technique, Linear prediction evolution algorithm, Mathematical inspired algorithm

## Abstract

Prediction-based evolutionary algorithm is one of the emerging category of meta-heuristic optimization techniques. The improved linear prediction evolution algorithm (ILPE) is a recently developed meta-heuristic optimization technique that draws inspiration from non-linear least-square fitting models. This article implements the concept of topological opposition-based learning, which was first applied in grey prediction evolutionary algorithms to the ILPE. In traditional evolutionary algorithms, after employing the mutation and crossover operator, it generates trial populations. The proposed algorithm constructs a new reproduction operator using the non-linear least square fitting model with topological opposition-based learning to generate trial individuals. This reproduction operator considers the population series as a time series and uses the topological opposition-based non-linear least square fitting model to predict the next generation of populations. The efficiency and accuracy of the algorithm are demonstrated through numerical experiments on CEC2014 and CEC2017 benchmark functions. The results of these experiments show that the proposed algorithm is highly effective in solving optimization problems.

• An improved linear prediction evolution algorithm based on topological opposition based learning (TILPE) is proposed.
• The proposed strategy treat the the population series as a time series.
• To validate the efficacy of TILPE, CEC2014 and CEC2017 benchmark functions are used.

## Graphical abstract

**Figure:**
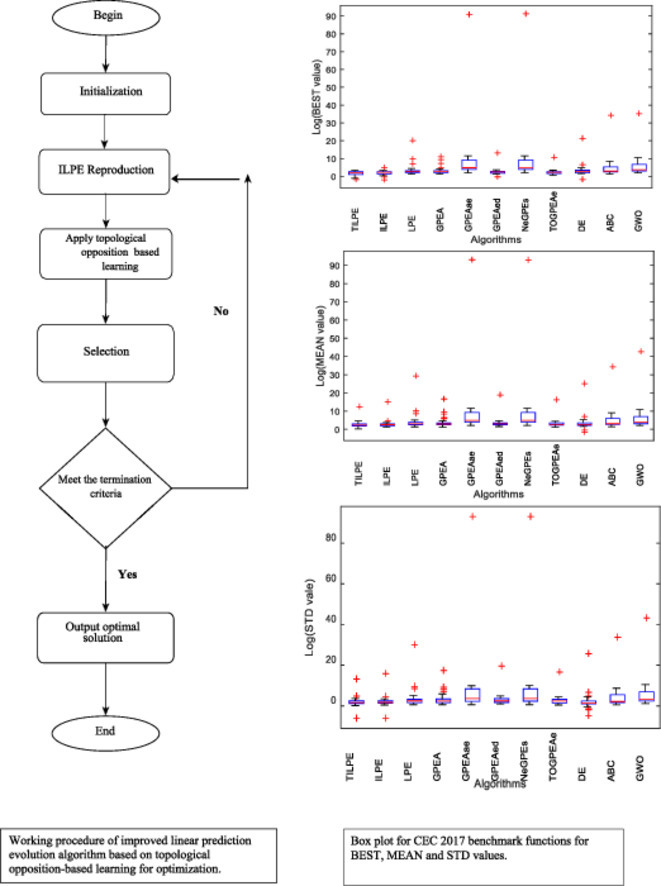

Specification table

**Table:** 

Subject Area:	Mathematics
More specific subject area:	Meta-heuristics Algorithm
Method name:	Mathematical inspired algorithm
Name and reference of original method:	An improved linear prediction evolution algorithm based on nonlinear least square fitting model for optimization. https://link.springer.com/article/10.1007/s00500-023-08500-6
Resource availability:	N/A

## Introduction

Meta-heuristic algorithms are well-known optimization approaches to deal with non-linear and complex problems [1]. However, the population-based methods are computationally costly due to the sluggish nature of the evolutionary process. Swarm intelligence has emerged as an important field in the area of nature-inspired techniques in recent times. It is mainly used to tackle real-world optimization problems. Based on the collective behavior of creatures that live in swarms or colonies. It uses the collaborative trial and error process to find a solution. The growing interest in meta-heuristic is completely justified by the limitations of gradient-based approaches and the development of machines with high processing capacity, which has enabled the establishment of more complicated meta-heuristics to handle NP-hard problems [2] effectively. Over the last few decades, a plethora of novel meta-heuristic algorithms have been developed and updated. The well-known meta-heuristic algorithms are particle swarm optimization (PSO) [3], ant colony optimization algorithm (ACO) [4], artificial bee colony algorithm (ABC) [5], moth-flame optimization (MFO) [6], cuckoo search optimization (CSO) [7], spider monkey optimization (SMO) [8], differential evolution (DE) [9], genetic algorithm (GA) [10], genetic programming (GP) [11], polar bear optimization (PBO) [12], whale optimization algorithm (WOA) [13], gravitational search algorithm (GSA) [14].

Metaheuristics inspired by the mathematical model have recently emerged. These metaheuristics operate differently from traditional methods and do not rely on mutation and crossover operators. Examples of such metaheuristics include the estimation of distribution algorithm (EDA) [15], grey prediction evolution algorithm (GPEA) [16], grey prediction evolution algorithm based on even difference grey model (GPEAed) [17], multivariable grey prediction evolution algorithm (MGPEA) [18], grey prediction evolution algorithm based on topological opposition-based learning (TOGPEA) [19], non-equidistant grey prediction evolution algorithm for global optimization (NeGPEs) [20], linear prediction evolution algorithm (LPE) [21], and improved linear prediction evolution algorithm (ILPE) [22]. These metaheuristics offer unique ways to solve optimization problems and are a promising area of research in evolutionary algorithms.

Unlike other metaheuristic algorithms, the improved linear prediction evolution algorithm (ILPE) is inspired by the non-linear least square fitting model [23], [24], [25]. The novel evolutionary algorithm treats the population series as a time series and utilizes the non-linear least square fitting model to predict the next-generation populations. ILPE has demonstrated promising results in optimizing CEC2014 [26] and CEC2017 [27] benchmark functions. It also differs from other evolutionary algorithms as it does not have the mutation and crossover operators, and its only parameter is the population size. This paper proposes an improved linear prediction evolution algorithm based on topological opposition-based learning (TILPE) to enhance its exploitation capability further.

The purpose of this study is to enhance the search mechanism of the original ILPE by incorporating the topological opposition-based learning (TOBL) strategy. The TOBL technique draws inspiration from opposition-based learning (OBL) proposed by Tizhoosh et al. [28]. Although many scholars have improved the OBL technique [29], [30], [31], [32], [33], [34], every OBL method requires at least one additional fitness function value to be computed. In contrast, TOBL generates candidate solutions by computing Manhattan distances between the best individual and all vertices of the hypercube. This helps the ILPE to avoid local minima stagnation and achieve faster convergence. The proposed technique’s performance has been evaluated on a set of 30-dimensional CEC2014 and CEC2017 benchmark functions, and the results have been compared with those of the original ILPE. The experimental results demonstrate that the proposed technique performs well compared to some state-of-the-art algorithms.

The article is organized as follows: An overview of the Improved Linear Prediction Evolution (ILPE) algorithm is given at the beginning of the article. The detailed presentation of the proposed Topological Opposition-Based ILPE followed. The article further includes the experimental results and various statistical analyses. Lastly, the paper presents concluding remarks.

## Preliminaries

**Algorithm 1:**
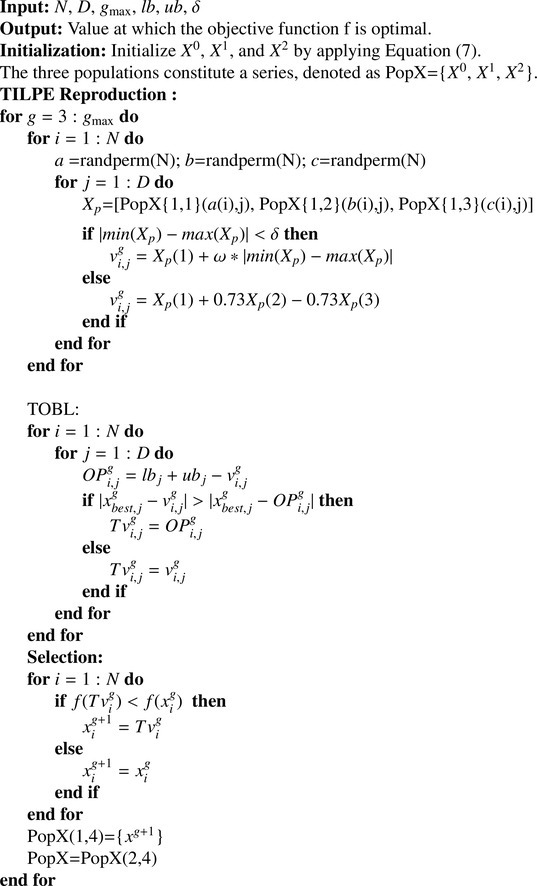
Pseudocode of TILPE

## Improved linear prediction evolution algorithm: ILPE [22]

The key innovation of the improved linear prediction evolution algorithm (ILPE) is its unique reproduction operator based on the non-linear least square fitting model. Unlike other meta-heuristic algorithms, ILPE does not rely on mutation and crossover operators to generate offspring. Instead, it employs the non-linear fitting operator as its sole reproduction operator.

### Mathematical model

This subsection describes the non-linear least square fitting model and the improved linear prediction operator.

### **Non-linear least square fitting model:**

Assume that we have n set of observations $\left(t_{1},x_{1}\right),\left(t_{2},x_{2}\right),\ldots ,\left(t_{n},x_{n}\right)$, the non-linear least square fitting technique can be described as follows:

(1) $$x_{i}=b_{0}+b_{1}\sin\left(\theta t_{i}\right)+b_{2}\cos\left(\theta t_{i}\right)+e_{i}.$$

Where $b_{0}$, $b_{1}$, and $b_{2}$, are unknown constants and $e_{i}$ is a random error component. And θ is set to be (5π/6) for ILPE. To find the value of $b_{0}$, $b_{1}$, and $b_{2}$ we use least square technique. The least square criteria is

(2) $$S=\sum_{i=1}^{n}e_{i}^{2}=\sum_{i=1}^{n}{\left(x_{i}-b_{0}-b_{1}\sin\left(\theta t_{i}\right)-b_{2}\cos\left(\theta t_{i}\right)\right)}^{2}.$$

The equation shown above is commonly referred to as the sum of the square loss [35], [36]. To determine the values of the unknown constants, we differentiate S partially with respect to $b_{0}$, $b_{1}$, and $b_{2}$ and set the results equal to zero, i.e.

(3) $$\left\{ \begin{array}{c} \frac{\partial S}{\partial b_{0}}=2*\sum_{i=1}^{n}\left(x_{i}-b_{0}-b_{1}\sin\left(\theta t_{i}\right)-b_{2}\cos\left(\theta t_{i}\right)\right)\left(-1\right)=0\\ \frac{\partial S}{\partial b_{1}}=2*\sum_{i=1}^{n}\left(x_{i}-b_{0}-b_{1}\sin\left(\theta t_{i}\right)-b_{2}\cos\left(\theta t_{i}\right)\right)\left(-\sin\left(\theta t_{i}\right)\right)=0\\ \frac{\partial S}{\partial b_{2}}=2*\sum_{i=1}^{n}\left(x_{i}-b_{0}-b_{1}\sin\left(\theta t_{i}\right)-b_{2}\cos\left(\theta t_{i}\right)\right)\left(-\cos\left(\theta t_{i}\right)\right)=0 \end{array}\right.$$

we can find the values of $b_{0}$, $b_{1}$ and $b_{2}$ from the following matrix.

(4) $$\left[\begin{array}{c} \sum_{i=1}^{n}x_{i}\\ \sum_{i=1}^{n}x_{i}\sin\left(\theta t_{i}\right)\\ \sum_{i=1}^{n}x_{i}\cos\left(\theta t_{i}\right) \end{array}\right]=\left[\begin{array}{ccc} n & \sum_{i=1}^{n}\sin\left(\theta t_{i}\right) & \sum_{i=1}^{n}\cos\left(\theta t_{i}\right)\\ \sum_{i=1}^{n}\sin\left(\theta t_{i}\right) & \sum_{i=1}^{n}\sin^{2}\left(\theta t_{i}\right) & \frac{\sum_{i=1}^{n}\sin\left(2\theta t_{i}\right)}{2}\\ \sum_{i=1}^{n}\cos\left(\theta t_{i}\right) & \frac{\sum_{i=1}^{n}\sin\left(2\theta t_{i}\right)}{2} & \sum_{i=1}^{n}\cos^{2}\left(\theta t_{i}\right) \end{array}\right]\left[\begin{array}{c} b_{0}\\ b_{1}\\ b_{2} \end{array}\right]$$

Let us assume the three input data points are $(x_{1}$, $x_{2}$, $x_{3})$. Using these data, we construct the fourth predicted data $\hat{x_{4}}$. Assume that the four data points are equally spaced and defined as $\left(1,x_{1}\right)$, $\left(2,x_{2}\right)$, $\left(3,x_{3}\right)$ and $\left(4,\hat{x_{4}}\right)$, respectively. As θ=5π/6 so we can get the value of $b_{0}$, $b_{1}$ and $b_{2}$ from Eq. (4).

(5) $$\left\{ \begin{array}{c} b_{0}=0.26796x_{1}+0.46412x_{2}+0.26796x_{3}\\ b_{1}=-0.26796x_{1}-.46412x_{2}+0.73205x_{3}\\ b_{2}=-1.0000x_{1}+0.26796x_{2}+0.73205x_{3} \end{array}\right.$$

By setting $e_{i}=0$ and substituting the values of $b_{0}$, $b_{1}$, and $b_{2}$ in Eq. (1), we can obtain the prediction for the fourth data point.

(6) $$\hat{x_{4}}=x_{1}+0.73x_{2}-0.73x_{3}$$

### Initialization

The ILPE randomly initializes the population having 3N individuals in the feasible region. We represents each population having N individuals by $x_{i}^{g}=\left(x_{i,1}^{g},x_{i,2}^{g},\ldots ,x_{i,D}^{g}\right)$ here, i=1,2,…N and $g=0,1,2,\ldots ,g_{\max}$, where g is the generation and $g_{\max}$ is the maximum number of generations. The top N of 3N individuals are considered as the first generation $X^{0}\left(g=0\right)$. Meanwhile, middle N of 3N individuals are considered as the second generation $X^{1}\left(g=1\right)$, and the bottom N of 3N individuals are assigned to be third generation $X^{2}\left(g=2\right)$. We initialize ith individuals in the jth dimension according to the following equation.

(7) $$x_{i,j}^{g}=lb_{j}+rand.\left(ub_{j}-lb_{j}\right).$$

Here, $ub_{j}$ and $lb_{j}$ are upper and lower bounds of the jth individual, respectively. And rand denotes, the uniformly distributed random number in the range [0,1].

### Reproduction

Based on the non-linear least square fitting model a novel reproduction operator was proposed in [22]. This operator forms the population series as a time series of the evolutionary algorithms. Let $X^{g-2},\;X^{g-1}$, and $X^{g}$, (g≥2) denotes the three successive population series, and three individuals $x_{r_{1}}^{g-2},\;\;x_{r_{2}}^{g-1}$, and $x_{r_{3}}^{g}$ are randomly chosen from $X^{g-2},\;X^{g-1}$, and $X^{g}$, respectively. Let the trial individuals are $v_{i}^{g}$ $=(v_{i,1}^{g},v_{i,2}^{g},\ldots ,v_{i,D}^{g}),i=1,2,\ldots N$.

Assume that M= $|min\left(X_{p}\right)-max\left(X_{p}\right)|$ where, $X_{p}$ is the population series.

Then the ILPE reproduction operator is

(8) $$v_{i,j}^{g}=\left\{ \begin{array}{c} x_{r_{1},j}^{g-2}+0.73x_{r_{2},j}^{g-1}-0.73x_{r_{3},j}^{g}\;if\;\delta <M\\ x_{r_{1},j}^{g-2}+\omega .M,\;otherwise \end{array}\right.$$

here δ belongs to [0.001, 0.1] is the forecasting parameter and ω is defined as follows

$\omega =(0.01-3.99\left(I-g_{\max}\right)/g_{\max})$.

Where I is the current iteration number and $g_{max}$ is the maximum number of generations.

### Selection operator

The selection operator keeps the good individuals from the previous generation who are deviating from the best outcome. According to the selection operator, a better person is always chosen for the following generation. Let the function f be minimized. Whether the newly generated individuals are passed on to the next iteration or not is determined by the greedy approach outlined below:

(9) $$\begin{array}{c} x_{i}^{g+1}=\left\{ \begin{array}{c} v_{i}^{g},\;\;\mathrm{if}\;f\left(v_{i}^{g}\right)<f\left(x_{i}^{g}\right)\\ x_{i}^{g},\;\;\;\;\;\;\;\;\;\;\mathrm{othe}\mathrm{rwise} \end{array}\right. \end{array}$$

## Proposed method

### Motivation

The balance between exploration and exploitation capabilities affects the meta-heuristic algorithm’s efficacy [37], significantly. It is preferable to explore the search space during the early iterations of the solution search process. This can be accomplished by enabling the candidate solutions to gain large step lengths in the initial iterations. In the later iterations, exploitation of a search space is required to avoid bypassing the global optima [38]. Therefore, the potential solution should have a small step length for the later iterations.

According to the reproduction Eq. (8) the generations coefficients plays a vital role in balancing exploitation and exploration. Whether it is a linear prediction evolution algorithm or improved linear prediction evolution algorithm the only change is the generations coefficients. Therefore, the effectiveness of these linear and improved linear prediction-based evolution algorithms depends upon the generations coefficients. The ILPE algorithms are successfully applied to CEC2014 and CEC2017 benchmark functions. Experimental analysis shows that it can explore the search efficiently through the non-linear least square fitting model. But its accuracy and convergence speed is not up to the mark. In order to accelerate the rate of convergence and solution accuracy of ILPE algorithm, this study propses ILPE with topological opposition based learning (TILPE). TILPE with better exploitation capacity, converges quickly.

### Topological opposition-based learning (TOBL)

The idea of TOBL was established in 2020 [19], and it is used to improve the capacity of searching and optimizing the meta-heuristic algorithms. It is mainly an improved version of OBL strategy, which has potent exploitation capability and less computational cost.

#### Topological opposite point

Let $x=(x_{i,1},x_{i,2},\ldots x_{i,D})$ be a point between [lb,ub] in a D-dimensional search space. The opposite point(OP) of x is expressed as follows: $OP=(OP_{i,1},OP_{i,2},\ldots OP_{i,D})$ where,

(10) $$OP_{i,j}=lb_{j}+ub_{j}-x_{i,j}\;\forall \;j=1,2,\ldots ,D.$$

And the topological opposite point Tx [19] of x is defined as $Tx=(Tx_{i,1},Tx_{i,2},\ldots Tx_{i,D})$. Where,

(11) $$\begin{array}{c} Tx_{i,j}=\left\{ \begin{array}{c} OP_{i,j},\;\mathrm{if}\;|x_{\mathrm{best},j}-x_{i,j}\left|>\right|x_{\mathrm{best},j}-OP_{i,j}|\\ x_{i,j},\;\;\;\;\;\;\;\;\;\;\;\;\;\;\;\mathrm{Othe}\mathrm{rwise} \end{array}\right. \end{array}$$

here $x_{best,j}$ is the jth dimension of the current best individual $x_{best}$. The Manhattan distance between the current best individual and all other potential solutions inside the feasible space is defined by the term $|x_{best,j}-x_{i,j}|$ and $|x_{best,j}-OP_{i,j}|$. That means the point Tx is the least Manhattan distance between the current best individual and all the possible solutions in the feasible space. This idea comes from opposition-based learning technique.

### Pseudocode

The original ILPE algorithm has only one parameter that is population size. It has strong exploration capability to search the global optima. The proposed TILPE algorithm applies TOBL strategy to improve its local search capacity so that it can attain the balance between exploration and exploitation strategy. Same as ILPE, TILPE initialize three populations using formula (7) and then generate the offspring by using the reproduction operator (Eq. (8)). Finally, select the candidate solution for subsequent generations by using a greedy selection mechanism (Eq. (9)). The pseudocode of TILPE is shown in Algorithm 1.

## Results and discussion

In order to analyze the efficiency and robustness of the proposed algorithm, TILPE is compared with ILPE [22] and other state of the art algorithms, including LPE [21], GPEA [16], GPEAae [39], GPEAed [17], NeGPEs [20], TOGPEA [19], DE [9], ABC [5] and GWO [6]. The CEC2014 [26]and CEC2017 [27] benchmark functions are used for all comparisons. These benchmark suits are divided into four categories functions such as unimodal, multimodal, hybrid, and composite functions.

### Experimental setting

The proposed TILPE is evaluated on a 30-dimensional search space in the range between [-100, 100]. For every function, 51 separate runs are performed for the population size 50. The termination criterion is set to the maximum number of generations which is taken as 5000. The DE algorithm uses DE/rand/1 mutation strategy where mutation and crossover rates are chosen to be 0.5, respectively. While the other experimental settings for the algorithms, ILPE, LPE [21], GPEA [16], GPEAae [39], GPEAed [17], NeGPEs [20], and TOGPEA [19] are similar to their original research article. The experiments were carried out on a 64-bit PC equipped with a 3.70 GHz Intel(R) Xeon(R) W-2135 CPU and 64 GB of RAM. MATLAB 2021a served as the platform for running these experiments. To maintain uniformity, the compared algorithms were re-implemented and executed on the exact computer configuration.

### Comparison of TILPE with other state of the art algorithms on CEC2014 benchmark functions

By using the experimental setting described in above, the performance of the proposed TILPE is compared with some variants of the GPEA algorithm and with a few other state-of-the-art meta-heuristic algorithms. The experimental results are shown in Table 1, including the five performance indicators, such as the minimum function value (BEST), the mean value (MEAN), the standard deviation value (STD), the median value (MED), and the maximum value (MAX) of the fitness error. Based on the reported results in Table 1, the performance of TILPE is examined. The best results obtained by each indicator in solving the CEC2014 benchmark functions are shown in bold. Moreover, the average rank of the compared algorithms is shown in Table 2. From Table 2, we can see that TILPE outperforms the other competitors evaluated in the best values with an average ranking of 1.9 while the average ranking of ILPE, LPE, GPEA, GPEAae, GPEAed, NeGPEs, TOGPEAe, DE, ABC, and GWO are 3.33, 8.1, 8.0, 4.27, 6.43, 4.57, 5.23, 5.2, 8.73 and 10.23. Also, the MEAN ranking of TILPE is 2.6 which is superior than all considered algorithms and STD ranking of TILPE is 4.4 which is only inferior than DE. Similarly the median ranking is superior than all other algorithms and the MAX ranking is only inferior than DE. Now, for the BEST solutions, TILPE gives better solutions for the functions $F_{4}$, $F_{8}-F_{10}$, $F_{13}$, $F_{15}$, $F_{18}$, $F_{20}$, $F_{22}-F_{26}$, $F_{29}$ and $F_{30}$ as compared to the other considered algorithms. TILPE also has the smallest mean (MEAN) for the functions $F_{9}$, $F_{10}$, $F_{13}$, $F_{15}$, $F_{19}$, $F_{20}$, $F_{23}-F_{25}$, $F_{29}$, $F_{30}$ and standard deviation (STD) values for the functions $F_{17}$, and $F_{30}$. Similarly for median and maximum values TILPE gives the better results for the functions $F_{9}$, $F_{10}$, $F_{13}$, $F_{14}$, $F_{20}$, $F_{24}-F_{26}$, $F_{29}$, $F_{30}$, and $F_{13}$, $F_{15}$, $F_{19}$, $F_{29}$. As for MEAN, TILPE performs moderately superior to DE but outperforms other competitors. For STD, it is inferior to DE and moderately superior to ILPE but outperforms other considered algorithms. Also, for MED it is comparable with DE but superior than other algorithm’s and for MAX values it is only inferior than ILPE and DE. It is evident from the above investigation that TILPE significantly outperforms GPEA and its variants in terms of solution accuracy, but its robustness is competitive with the DE algorithm.

**Table 1:** Results of TILPE with other metaheuristic algorithms on CEC2014 benchmark functions

Function	Algorithm	BEST	MEAN	STD	MED	MAX

$F_{1}$	TILPE	2.876E+04	2.321E+05	1.768E+05	1.840E+05	1.067E+06
	ILPE	**2.336E+04**	**1.687E+05**	**9.699E+04**	**1.696E+05**	**4.491E+05**
	LPE	2.222E+07	8.388E+07	4.097E+07	7.573E+07	2.091E+08
	GPEA	6.502E+07	1.304E+08	3.841E+07	1.229E+08	2.437E+08
	GPEAae	2.367E+05	1.444E+06	9.887E+05	1.155E+06	5.255E+06
	GPEAed	3.501E+05	1.286E+06	7.933E+05	9.837E+05	4.043E+06
	NeGPEs	9.606E+04	7.923E+05	5.952E+05	6.285E+05	3.812E+06
	TOGPEAe	2.323E+05	1.184E+06	8.273E+05	9.771E+05	3.778E+06
	DE	3.508E+06	9.320E+06	2.870E+06	9.195E+06	1.659E+07
	ABC	4.198E+07	9.723E+07	2.594E+07	9.868E+07	1.558E+08
	GWO	1.284E+09	2.582E+09	8.702E+08	2.368E+09	4.640E+09
$F_{2}$	TILPE	1.865E-02	5.556E+02	1.930E+03	2.674E-01	8.116E+03
	ILPE	1.975E-02	5.414E+02	3.864E+03	1.936E-01	2.760E+04
	LPE	1.013E+10	1.678E+10	4.258E+09	1.614E+10	2.661E+10
	GPEA	4.094E+09	7.176E+09	1.887E+09	7.050E+09	1.239E+10
	GPEAae	5.814E+02	3.501E+03	2.816E+03	2.919E+03	1.706E+04
	GPEAed	1.722E+00	6.502E+02	3.209E+03	4.754E+01	2.208E+04
	NeGPEs	6.589E-03	1.757E+01	4.744E+01	1.670E+00	2.853E+02
	TOGPEAe	4.214E+00	2.399E+03	4.962E+03	6.697E+02	2.589E+04
	DE	**0.000E+00**	**9.474E-15**	**1.353E-14**	**0.000E+00**	**2.842E-14**
	ABC	5.391E+08	1.829E+09	5.866E+08	1.758E+09	3.396E+09
	GWO	7.091E+10	1.446E+11	3.234E+10	1.428E+11	2.391E+11
$F_{3}$	TILPE	8.637E-02	9.868E+00	9.683E+00	6.486E+00	3.996E+01
	ILPE	6.722E-01	1.033E+01	1.019E+01	6.732E+00	4.399E+01
	LPE	9.782E+03	1.884E+04	4.811E+03	1.822E+04	2.978E+04
	GPEA	8.485E+03	1.710E+04	6.225E+03	1.637E+04	3.094E+04
	GPEAae	3.501E+00	1.675E+03	1.698E+03	1.177E+03	8.691E+03
	GPEAed	1.443E+00	2.333E+03	3.736E+03	1.578E+03	2.422E+04
	NeGPEs	1.687E-01	7.513E+02	1.419E+03	1.186E+02	6.381E+03
	TOGPEAe	4.054E+00	1.694E+03	2.118E+03	9.264E+02	9.341E+03
	DE	**0.000E+00**	**2.452E-14**	**2.843E-14**	**0.000E+00**	**5.684E-14**
	ABC	3.281E+03	9.307E+03	3.239E+03	9.259E+03	1.785E+04
	GWO	2.040E+05	3.649E+05	6.013E+04	3.686E+05	5.040E+05
$F_{4}$	TILPE	**4.319E-03**	4.065E+01	3.855E+01	6.763E+01	1.621E+02
	ILPE	8.412E-03	4.010E+01	3.841E+01	6.766E+01	1.341E+02
	LPE	5.595E+02	1.420E+03	5.181E+02	1.343E+03	2.922E+03
	GPEA	3.582E+02	7.733E+02	2.487E+02	7.415E+02	1.740E+03
	GPEAae	1.598E-01	9.134E+01	4.271E+01	8.250E+01	1.917E+02
	GPEAed	2.089E+00	1.057E+02	4.600E+01	9.013E+01	1.995E+02
	NeGPEs	8.669E-03	8.744E+01	3.622E+01	7.524E+01	1.534E+02
	TOGPEAe	1.962E+01	1.282E+02	4.129E+01	1.275E+02	2.412E+02
	DE	2.457E-01	**2.247E+00**	**9.501E+00**	**9.284E-01**	**6.872E+01**
	ABC	2.728E+02	3.872E+02	6.771E+01	3.856E+02	5.244E+02
	GWO	1.556E+04	3.649E+04	1.496E+04	3.218E+04	7.962E+04
$F_{5}$	TILPE	2.000E+01	2.066E+01	3.533E-01	2.085E+01	2.102E+01
	ILPE	**2.000E+01**	**2.005E+01**	9.345E-02	**2.000E+01**	**2.050E+01**
	LPE	2.072E+01	2.094E+01	8.821E-02	2.097E+01	2.110E+01
	GPEA	2.079E+01	2.101E+01	7.744E-02	2.102E+01	2.112E+01
	GPEAae	2.000E+01	2.100E+01	1.583E-01	2.104E+01	2.115E+01
	GPEAed	2.082E+01	2.107E+01	1.081E-01	2.108E+01	2.124E+01
	NeGPEs	2.001E+01	2.080E+01	2.424E-01	2.091E+01	2.103E+01
	TOGPEAe	2.004E+01	2.059E+01	4.020E-01	2.047E+01	2.107E+01
	DE	2.078E+01	2.094E+01	5.133E-02	2.095E+01	2.102E+01
	ABC	2.042E+01	2.054E+01	**3.697E-02**	2.054E+01	2.061E+01
	GWO	2.031E+01	2.059E+01	1.010E-01	2.060E+01	2.080E+01
$F_{6}$	TILPE	9.005E-01	4.025E+00	1.996E+00	3.855E+00	9.853E+00
	ILPE	6.710E+00	1.591E+01	3.803E+00	1.551E+01	2.533E+01
	LPE	1.853E+01	2.158E+01	1.632E+00	2.169E+01	2.566E+01
	GPEA	1.753E+01	2.204E+01	2.073E+00	2.211E+01	2.739E+01
	GPEAae	5.197E+00	1.027E+01	2.775E+00	1.002E+01	1.617E+01
	GPEAed	1.561E+01	2.741E+01	6.571E+00	2.626E+01	4.387E+01
	NeGPEs	4.743E+00	1.282E+01	3.576E+00	1.294E+01	1.983E+01
	TOGPEAe	1.022E+01	1.804E+01	3.360E+00	1.787E+01	2.602E+01
	DE	**0.000E+00**	**7.059E-03**	**4.314E-02**	**0.000E+00**	**3.042E-01**
	ABC	2.167E+01	2.487E+01	1.283E+00	2.504E+01	2.689E+01
	GWO	1.753E+01	2.620E+01	3.223E+00	2.603E+01	3.415E+01
$F_{7}$	TILPE	6.381E-10	2.754E-03	4.820E-03	2.688E-08	1.724E-02
	ILPE	1.894E-09	2.615E-03	1.396E-02	3.702E-08	9.886E-02
	LPE	8.323E+01	1.504E+02	4.170E+01	1.510E+02	2.557E+02
	GPEA	2.470E+01	6.575E+01	2.193E+01	6.412E+01	1.323E+02
	GPEAae	5.735E-07	1.742E-03	5.593E-03	3.031E-06	3.441E-02
	GPEAed	4.127E-11	1.184E-02	1.282E-02	1.232E-02	5.171E-02
	NeGPEs	3.070E-12	8.779E-03	1.304E-02	5.311E-10	7.123E-02
	TOGPEAe	6.812E-06	5.928E-02	5.388E-02	4.853E-02	3.166E-01
	DE	**0.000E+00**	**2.900E-04**	**1.450E-03**	**0.000E+00**	**7.396E-03**
	ABC	9.933E+00	2.834E+01	7.587E+00	2.825E+01	4.296E+01
	GWO	1.321E+03	1.971E+03	2.965E+02	1.934E+03	2.610E+03
$F_{8}$	TILPE	**1.094E+01**	4.430E+01	1.419E+01	4.294E+01	7.822E+01
	ILPE	3.184E+01	6.260E+01	1.844E+01	6.268E+01	1.088E+02
	LPE	1.116E+02	1.453E+02	2.050E+01	1.442E+02	1.948E+02
	GPEA	5.058E+01	8.325E+01	1.447E+01	8.209E+01	1.125E+02
	GPEAae	3.184E+01	6.420E+01	1.533E+01	6.268E+01	1.025E+02
	GPEAed	4.577E+01	8.921E+01	3.831E+01	8.159E+01	2.973E+02
	NeGPEs	2.388E+01	**4.220E+01**	1.023E+01	**4.079E+01**	**7.562E+01**
	TOGPEAe	1.691E+01	4.596E+01	1.384E+01	4.776E+01	7.562E+01
	DE	1.079E+02	1.268E+02	**8.325E+00**	1.278E+02	1.481E+02
	ABC	6.944E+01	9.280E+01	9.146E+00	9.372E+01	1.086E+02
	GWO	3.435E+02	4.802E+02	7.253E+01	4.807E+02	6.422E+02
$F_{9}$	TILPE	**1.492E+01**	**4.473E+01**	1.917E+01	**4.079E+01**	9.402E+01
	ILPE	3.482E+01	6.478E+01	1.599E+01	6.268E+01	1.134E+02
	LPE	1.069E+02	1.432E+02	2.231E+01	1.422E+02	2.015E+02
	GPEA	5.081E+01	9.041E+01	1.996E+01	8.590E+01	1.432E+02
	GPEAae	4.776E+01	7.552E+01	1.862E+01	6.965E+01	1.323E+02
	GPEAed	6.169E+01	1.042E+02	3.876E+01	9.850E+01	3.247E+02
	NeGPEs	2.487E+01	4.686E+01	1.207E+01	4.676E+01	**7.761E+01**
	TOGPEAe	1.691E+01	5.041E+01	1.512E+01	4.875E+01	8.557E+01
	DE	1.575E+02	1.799E+02	**9.918E+00**	1.802E+02	1.979E+02
	ABC	1.813E+02	2.232E+02	1.501E+01	2.234E+02	2.522E+02
	GWO	3.449E+02	6.392E+02	1.143E+02	6.171E+02	9.779E+02
$F_{10}$	TILPE	**5.165E+02**	**1.273E+03**	5.240E+02	**1.183E+03**	3.228E+03
	ILPE	1.778E+03	3.399E+03	6.814E+02	3.389E+03	4.996E+03
	LPE	4.231E+03	6.469E+03	7.560E+02	6.607E+03	7.739E+03
	GPEA	7.651E+02	1.850E+03	4.985E+02	1.817E+03	3.110E+03
	GPEAae	8.551E+02	2.750E+03	1.419E+03	2.480E+03	8.220E+03
	GPEAed	1.979E+03	6.884E+03	1.889E+03	7.625E+03	8.985E+03
	NeGPEs	6.533E+02	1.680E+03	5.008E+02	1.724E+03	3.262E+03
	TOGPEAe	1.591E+03	3.659E+03	1.822E+03	2.937E+03	8.117E+03
	DE	3.514E+03	4.670E+03	3.605E+02	4.686E+03	5.233E+03
	ABC	1.091E+03	2.091E+03	**2.551E+02**	2.124E+03	**2.497E+03**
	GWO	4.990E+03	5.977E+03	5.237E+02	5.960E+03	7.033E+03
$F_{11}$	TILPE	2.160E+03	5.423E+03	1.485E+03	5.894E+03	7.229E+03
	ILPE	2.152E+03	3.561E+03	7.060E+02	3.585E+03	5.383E+03
	LPE	5.910E+03	6.991E+03	4.938E+02	7.013E+03	7.819E+03
	GPEA	1.961E+03	**2.982E+03**	1.032E+03	**2.702E+03**	6.971E+03
	GPEAae	**1.874E+03**	7.161E+03	1.148E+03	7.451E+03	7.919E+03
	GPEAed	4.275E+03	7.863E+03	8.030E+02	8.138E+03	8.782E+03
	NeGPEs	1.930E+03	3.389E+03	9.548E+02	3.233E+03	7.018E+03
	TOGPEAe	2.346E+03	5.376E+03	1.735E+03	4.890E+03	7.951E+03
	DE	6.134E+03	6.859E+03	**2.625E+02**	6.883E+03	7.302E+03
	ABC	3.433E+03	4.156E+03	2.658E+02	4.147E+03	**4.618E+03**
	GWO	3.619E+03	4.915E+03	5.307E+02	4.822E+03	6.116E+03
$F_{12}$	TILPE	4.078E-02	1.263E+00	6.249E-01	1.210E+00	2.776E+00
	ILPE	**1.618E-03**	**5.509E-02**	9.444E-02	**2.246E-02**	**5.753E-01**
	LPE	9.600E-01	2.158E+00	5.532E-01	2.143E+00	3.308E+00
	GPEA	9.858E-01	2.821E+00	4.263E-01	2.852E+00	3.563E+00
	GPEAae	4.155E-02	2.656E+00	8.633E-01	2.857E+00	3.737E+00
	GPEAed	1.206E-01	2.822E+00	1.099E+00	2.674E+00	4.755E+00
	NeGPEs	1.576E-02	5.388E-01	8.075E-01	1.587E-01	2.678E+00
	TOGPEAe	2.824E-02	1.181E+00	1.281E+00	2.918E-01	3.356E+00
	DE	1.709E+00	2.421E+00	2.379E-01	2.472E+00	2.862E+00
	ABC	6.415E-01	8.524E-01	**8.652E-02**	8.566E-01	1.012E+00
	GWO	2.172E-01	7.255E-01	3.543E-01	6.360E-01	1.884E+00
$F_{13}$	TILPE	**1.205E-01**	**2.374E-01**	5.356E-02	**2.339E-01**	**3.493E-01**
	ILPE	2.561E-01	4.507E-01	1.106E-01	4.453E-01	7.312E-01
	LPE	1.487E+00	3.220E+00	6.074E-01	3.293E+00	4.381E+00
	GPEA	2.631E-01	1.161E+00	8.088E-01	7.142E-01	2.547E+00
	GPEAae	2.501E-01	4.945E-01	1.397E-01	5.060E-01	7.613E-01
	GPEAed	2.101E-01	4.847E-01	1.386E-01	4.745E-01	9.141E-01
	NeGPEs	2.574E-01	4.257E-01	9.581E-02	4.165E-01	6.163E-01
	TOGPEAe	1.932E-01	4.696E-01	1.287E-01	4.731E-01	8.461E-01
	DE	1.918E-01	3.468E-01	**4.550E-02**	3.538E-01	4.264E-01
	ABC	4.450E-01	7.356E-01	1.104E-01	7.175E-01	1.073E+00
	GWO	7.308E+00	1.057E+01	1.517E+00	1.016E+01	1.455E+01
$F_{14}$	TILPE	1.510E-01	2.456E-01	8.632E-02	**2.271E-01**	7.598E-01
	ILPE	**1.136E-01**	**2.355E-01**	5.612E-02	2.313E-01	3.590E-01
	LPE	2.117E+01	5.718E+01	1.684E+01	5.987E+01	9.346E+01
	GPEA	1.209E+01	2.422E+01	6.572E+00	2.362E+01	4.152E+01
	GPEAae	1.902E-01	2.961E-01	5.799E-02	2.865E-01	4.774E-01
	GPEAed	1.897E-01	2.769E-01	6.277E-02	2.598E-01	4.734E-01
	NeGPEs	2.020E-01	3.108E-01	5.809E-02	3.069E-01	4.213E-01
	TOGPEAe	1.803E-01	2.837E-01	9.998E-02	2.708E-01	9.089E-01
	DE	1.505E-01	2.636E-01	**3.271E-02**	2.666E-01	**3.277E-01**
	ABC	7.000E-01	8.173E+00	4.203E+00	8.121E+00	1.633E+01
	GWO	5.751E+02	8.025E+02	1.246E+02	7.990E+02	1.084E+03
$F_{15}$	TILPE	**2.535E+00**	**4.801E+00**	2.796E+00	5.859E+00	**1.441E+01**
	ILPE	4.876E+00	1.231E+01	3.853E+00	1.250E+01	2.133E+01
	LPE	1.511E+02	1.606E+03	1.704E+03	1.131E+03	9.574E+03
	GPEA	2.900E+01	1.903E+02	1.628E+02	1.440E+02	6.749E+02
	GPEAae	3.043E+00	1.189E+01	6.457E+00	1.337E+01	2.417E+01
	GPEAed	9.188E+00	2.652E+01	1.107E+01	2.311E+01	6.242E+01
	NeGPEs	3.042E+00	5.889E+00	2.589E+00	**5.049E+00**	1.522E+01
	TOGPEAe	6.496E+00	2.141E+01	7.683E+00	2.181E+01	4.303E+01
	DE	1.338E+01	1.596E+01	**9.671E-01**	1.591E+01	1.775E+01
	ABC	4.173E+01	1.807E+02	1.151E+02	1.539E+02	5.978E+02
	GWO	1.721E+07	1.073E+08	7.351E+07	8.969E+07	3.730E+08
$F_{16}$	TILPE	9.742E+00	1.182E+01	8.130E-01	1.200E+01	1.295E+01
	ILPE	1.014E+01	1.203E+01	7.260E-01	1.225E+01	1.299E+01
	LPE	1.150E+01	1.259E+01	4.414E-01	1.272E+01	1.311E+01
	GPEA	1.048E+01	1.191E+01	7.880E-01	1.222E+01	1.327E+01
	GPEAae	**8.943E+00**	1.244E+01	1.078E+00	1.288E+01	1.346E+01
	GPEAed	1.124E+01	1.340E+01	4.629E-01	1.354E+01	1.385E+01
	NeGPEs	1.001E+01	**1.161E+01**	7.705E-01	**1.182E+01**	1.266E+01
	TOGPEAe	1.005E+01	1.206E+01	9.032E-01	1.218E+01	1.331E+01
	DE	1.203E+01	1.246E+01	**1.977E-01**	1.249E+01	1.285E+01
	ABC	1.128E+01	1.188E+01	2.433E-01	1.187E+01	**1.225E+01**
	GWO	1.156E+01	1.262E+01	4.016E-01	1.258E+01	1.342E+01
$F_{17}$	TILPE	1.508E+03	1.894E+03	**3.280E+02**	1.858E+03	3.885E+03
	ILPE	**5.693E+02**	**1.070E+03**	3.347E+02	**1.014E+03**	**2.072E+03**
	LPE	1.222E+03	6.025E+04	9.236E+04	3.345E+04	5.849E+05
	GPEA	1.356E+05	1.782E+06	1.212E+06	1.624E+06	5.443E+06
	GPEAae	3.743E+03	2.305E+04	2.150E+04	1.577E+04	1.196E+05
	GPEAed	4.905E+03	4.171E+04	3.814E+04	3.010E+04	1.720E+05
	NeGPEs	3.576E+03	1.863E+04	1.287E+04	1.333E+04	5.815E+04
	TOGPEAe	2.143E+03	2.374E+04	1.990E+04	1.842E+04	9.241E+04
	DE	2.412E+03	1.437E+04	1.873E+04	7.030E+03	9.039E+04
	ABC	3.653E+06	7.395E+06	2.432E+06	7.167E+06	1.453E+07
	GWO	7.888E+07	1.976E+08	7.888E+07	1.825E+08	4.381E+08
$F_{18}$	TILPE	**1.236E+01**	3.273E+02	5.865E+02	1.674E+02	3.668E+03
	ILPE	4.209E+01	2.135E+02	9.091E+01	2.225E+02	3.936E+02
	LPE	6.806E+01	1.621E+02	8.188E+01	1.482E+02	5.895E+02
	GPEA	1.577E+02	5.515E+02	4.340E+02	3.835E+02	1.902E+03
	GPEAae	2.917E+01	3.760E+03	2.928E+03	3.422E+03	1.305E+04
	GPEAed	6.629E+01	2.527E+03	3.292E+03	1.048E+03	1.849E+04
	NeGPEs	6.726E+01	4.474E+03	3.231E+03	4.329E+03	1.113E+04
	TOGPEAe	1.857E+01	2.554E+03	2.695E+03	1.813E+03	9.161E+03
	DE	7.050E+01	**8.817E+01**	**8.731E+00**	**8.734E+01**	**1.109E+02**
	ABC	4.981E+06	3.018E+07	1.680E+07	2.857E+07	7.972E+07
	GWO	4.563E+09	5.775E+09	2.905E+09	5.128E+09	2.477E+10
$F_{19}$	TILPE	2.455E+00	**4.073E+00**	6.246E-01	4.203E+00	**5.559E+00**
	ILPE	**1.607E+00**	4.126E+00	1.277E+00	**3.722E+00**	7.540E+00
	LPE	8.592E+00	4.058E+01	3.192E+01	2.473E+01	1.785E+02
	GPEA	1.272E+01	6.403E+01	3.117E+01	6.377E+01	1.479E+02
	GPEAae	3.500E+00	9.857E+00	1.406E+01	6.401E+00	6.732E+01
	GPEAed	8.094E+00	2.629E+01	2.880E+01	1.172E+01	1.305E+02
	NeGPEs	2.728E+00	1.106E+01	1.578E+01	6.582E+00	6.541E+01
	TOGPEAe	4.571E+00	1.165E+01	1.157E+01	9.323E+00	6.760E+01
	DE	4.450E+00	5.276E+00	**4.220E-01**	5.256E+00	6.117E+00
	ABC	2.816E+01	6.630E+01	1.602E+01	6.676E+01	1.009E+02
	GWO	5.320E+02	8.891E+02	4.324E+02	7.519E+02	2.780E+03
$F_{20}$	TILPE	**1.611E+01**	**4.377E+01**	1.084E+01	**4.376E+01**	6.847E+01
	ILPE	2.120E+01	5.235E+01	2.254E+01	4.838E+01	1.283E+02
	LPE	5.404E+01	9.743E+02	1.573E+03	2.937E+02	6.671E+03
	GPEA	6.533E+02	6.747E+03	4.024E+03	6.045E+03	1.674E+04
	GPEAae	5.824E+01	3.822E+02	4.364E+02	2.449E+02	2.127E+03
	GPEAed	6.752E+01	8.554E+02	1.022E+03	3.707E+02	4.405E+03
	NeGPEs	4.149E+01	5.077E+02	6.474E+02	3.173E+02	3.345E+03
	TOGPEAe	2.745E+01	2.909E+02	2.715E+02	2.020E+02	1.465E+03
	DE	3.829E+01	5.048E+01	**4.948E+00**	5.007E+01	**6.220E+01**
	ABC	2.339E+03	8.147E+03	3.068E+03	7.988E+03	1.423E+04
	GWO	1.324E+05	3.685E+06	5.152E+06	1.553E+06	2.293E+07
$F_{21}$	TILPE	5.430E+02	1.157E+03	2.557E+02	1.210E+03	1.720E+03
	ILPE	**1.470E+02**	**6.213E+02**	**2.134E+02**	**6.345E+02**	**1.158E+03**
	LPE	3.998E+02	2.851E+03	3.267E+03	1.678E+03	1.540E+04
	GPEA	1.358E+04	1.577E+05	1.229E+05	1.200E+05	5.253E+05
	GPEAae	7.067E+02	1.718E+04	1.653E+04	1.100E+04	6.223E+04
	GPEAed	1.005E+03	1.351E+04	1.270E+04	8.620E+03	5.355E+04
	NeGPEs	7.810E+02	8.332E+03	7.252E+03	5.660E+03	3.163E+04
	TOGPEAe	3.487E+02	6.805E+03	7.597E+03	4.029E+03	3.057E+04
	DE	8.271E+02	1.382E+03	2.983E+02	1.334E+03	2.380E+03
	ABC	6.536E+05	1.783E+06	6.802E+05	1.637E+06	3.428E+06
	GWO	1.205E+08	1.559E+08	4.471E+07	1.417E+08	4.045E+08
$F_{22}$	TILPE	**2.502E+01**	4.102E+02	1.863E+02	4.594E+02	7.247E+02
	ILPE	1.415E+02	4.505E+02	1.903E+02	4.588E+02	9.135E+02
	LPE	3.226E+01	2.870E+02	2.060E+02	2.034E+02	7.456E+02
	GPEA	1.480E+02	3.965E+02	1.600E+02	4.447E+02	8.909E+02
	GPEAae	6.421E+01	4.060E+02	2.180E+02	3.714E+02	9.817E+02
	GPEAed	2.838E+02	9.153E+02	3.051E+02	9.043E+02	1.478E+03
	NeGPEs	2.941E+02	6.411E+02	2.044E+02	6.467E+02	1.080E+03
	TOGPEAe	2.226E+02	6.513E+02	1.986E+02	6.568E+02	9.960E+02
	DE	2.549E+01	**1.041E+02**	**1.051E+02**	**4.566E+01**	**4.059E+02**
	ABC	2.977E+02	5.917E+02	1.072E+02	6.089E+02	7.874E+02
	GWO	3.339E+03	6.583E+03	4.869E+03	3.922E+03	2.094E+04
$F_{23}$	TILPE	**3.152E+02**	**3.152E+02**	8.575E-07	3.152E+02	3.152E+02
	ILPE	3.152E+02	3.152E+02	5.611E-06	3.152E+02	3.152E+02
	LPE	3.274E+02	3.612E+02	2.341E+01	3.593E+02	4.641E+02
	GPEA	3.350E+02	3.565E+02	1.246E+01	3.549E+02	3.866E+02
	GPEAae	3.152E+02	3.152E+02	1.113E-04	3.152E+02	3.152E+02
	GPEAed	3.152E+02	3.153E+02	5.162E-02	3.153E+02	3.155E+02
	NeGPEs	3.152E+02	3.152E+02	1.434E-07	3.152E+02	3.152E+02
	TOGPEAe	3.152E+02	3.153E+02	7.276E-02	3.153E+02	3.156E+02
	DE	3.152E+02	3.152E+02	**0.000E+00**	**3.152E+02**	**3.152E+02**
	ABC	3.250E+02	3.459E+02	8.060E+00	3.476E+02	3.659E+02
	GWO	7.491E+02	1.104E+03	2.005E+02	1.056E+03	1.596E+03
$F_{24}$	TILPE	**2.000E+02**	**2.109E+02**	1.219E+01	**2.002E+02**	2.335E+02
	ILPE	2.236E+02	2.367E+02	8.316E+00	2.396E+02	2.504E+02
	LPE	2.477E+02	2.613E+02	5.805E+00	2.618E+02	2.732E+02
	GPEA	2.321E+02	2.456E+02	4.734E+00	2.460E+02	2.573E+02
	GPEAae	2.238E+02	2.343E+02	8.411E+00	2.356E+02	2.486E+02
	GPEAed	2.291E+02	2.433E+02	6.486E+00	2.445E+02	2.551E+02
	NeGPEs	2.250E+02	2.378E+02	7.773E+00	2.397E+02	2.555E+02
	TOGPEAe	2.360E+02	2.514E+02	5.351E+00	2.521E+02	2.632E+02
	DE	2.002E+02	2.215E+02	4.409E+00	2.220E+02	**2.243E+02**
	ABC	2.563E+02	2.640E+02	**3.363E+00**	2.638E+02	2.723E+02
	GWO	3.320E+02	4.998E+02	8.161E+01	4.877E+02	7.067E+02
$F_{25}$	TILPE	**2.000E+02**	**2.004E+02**	1.179E+00	**2.000E+02**	2.045E+02
	ILPE	2.027E+02	2.085E+02	3.468E+00	2.092E+02	2.147E+02
	LPE	2.110E+02	2.160E+02	2.265E+00	2.164E+02	2.212E+02
	GPEA	2.090E+02	2.136E+02	2.184E+00	2.132E+02	2.190E+02
	GPEAae	2.032E+02	2.089E+02	4.274E+00	2.099E+02	2.180E+02
	GPEAed	2.037E+02	2.157E+02	4.425E+00	2.158E+02	2.233E+02
	NeGPEs	2.005E+02	2.010E+02	**2.313E-01**	2.010E+02	**2.015E+02**
	TOGPEAe	2.037E+02	2.084E+02	3.826E+00	2.079E+02	2.194E+02
	DE	2.049E+02	2.068E+02	1.084E+00	2.069E+02	2.092E+02
	ABC	2.113E+02	2.172E+02	2.346E+00	2.171E+02	2.225E+02
	GWO	2.157E+02	2.636E+02	2.793E+01	2.532E+02	3.387E+02
$F_{26}$	TILPE	**1.001E+02**	1.081E+02	2.709E+01	**1.002E+02**	2.000E+02
	ILPE	1.002E+02	1.004E+02	1.156E-01	1.004E+02	1.008E+02
	LPE	1.003E+02	1.017E+02	8.326E-01	1.019E+02	1.031E+02
	GPEA	1.003E+02	1.272E+02	4.398E+01	1.007E+02	2.005E+02
	GPEAae	1.002E+02	1.005E+02	1.280E-01	1.005E+02	1.008E+02
	GPEAed	1.002E+02	1.005E+02	1.300E-01	1.005E+02	1.008E+02
	NeGPEs	1.003E+02	1.024E+02	1.395E+01	1.004E+02	2.001E+02
	TOGPEAe	1.002E+02	1.024E+02	1.396E+01	1.005E+02	2.001E+02
	DE	1.003E+02	**1.003E+02**	**3.748E-02**	1.003E+02	**1.004E+02**
	ABC	1.006E+02	1.009E+02	1.990E-01	1.009E+02	1.018E+02
	GWO	2.422E+02	6.902E+02	4.208E+02	4.437E+02	1.478E+03
$F_{27}$	TILPE	3.314E+02	4.006E+02	2.399E+01	4.008E+02	4.888E+02
	ILPE	4.004E+02	4.319E+02	8.784E+01	4.007E+02	7.519E+02
	LPE	4.163E+02	8.077E+02	1.296E+02	8.271E+02	9.542E+02
	GPEA	4.724E+02	8.931E+02	1.004E+02	9.036E+02	1.038E+03
	GPEAae	3.579E+02	5.659E+02	8.281E+01	5.763E+02	7.665E+02
	GPEAed	4.017E+02	8.202E+02	2.435E+02	8.965E+02	1.373E+03
	NeGPEs	5.481E+02	7.340E+02	9.329E+01	7.383E+02	9.430E+02
	TOGPEAe	6.429E+02	8.398E+02	9.007E+01	8.362E+02	1.041E+03
	DE	**3.000E+02**	**3.023E+02**	**1.553E+01**	**3.000E+02**	**4.108E+02**
	ABC	3.813E+02	4.749E+02	2.190E+01	4.803E+02	5.194E+02
	GWO	9.532E+02	1.157E+03	8.638E+01	1.166E+03	1.324E+03
$F_{28}$	TILPE	7.599E+02	9.473E+02	6.974E+01	9.584E+02	1.074E+03
	ILPE	9.757E+02	1.530E+03	4.293E+02	1.429E+03	3.099E+03
	LPE	1.254E+03	1.689E+03	2.245E+02	1.676E+03	2.664E+03
	GPEA	1.118E+03	1.639E+03	2.038E+02	1.649E+03	2.302E+03
	GPEAae	7.731E+02	1.068E+03	1.617E+02	1.043E+03	1.551E+03
	GPEAed	1.166E+03	2.275E+03	1.003E+03	1.856E+03	4.608E+03
	NeGPEs	1.120E+03	1.418E+03	1.608E+02	1.420E+03	1.981E+03
	TOGPEAe	9.887E+02	1.309E+03	1.991E+02	1.344E+03	1.863E+03
	DE	**6.456E+02**	**7.911E+02**	**4.848E+01**	**7.847E+02**	**9.674E+02**
	ABC	1.170E+03	1.449E+03	1.307E+02	1.465E+03	1.745E+03
	GWO	7.895E+02	1.242E+03	2.900E+02	1.212E+03	2.053E+03
$F_{29}$	TILPE	**3.216E+02**	**7.251E+02**	2.099E+02	**6.891E+02**	**1.109E+03**
	ILPE	3.801E+02	8.890E+02	1.922E+02	9.004E+02	1.384E+03
	LPE	8.639E+02	9.304E+04	5.981E+05	2.055E+03	4.271E+06
	GPEA	1.065E+03	4.419E+04	1.203E+05	1.848E+03	6.851E+05
	GPEAae	9.564E+02	2.034E+03	1.217E+03	1.554E+03	6.827E+03
	GPEAed	9.230E+02	1.832E+03	5.583E+02	1.803E+03	2.886E+03
	NeGPEs	1.463E+03	1.671E+05	1.176E+06	2.572E+03	8.403E+06
	TOGPEAe	1.347E+03	1.673E+05	1.178E+06	2.476E+03	8.417E+06
	DE	9.929E+02	1.372E+03	**1.527E+02**	1.350E+03	1.704E+03
	ABC	1.071E+05	4.041E+05	1.444E+05	4.040E+05	8.128E+05
	GWO	2.434E+06	6.309E+06	2.930E+06	5.488E+06	1.451E+07
$F_{30}$	TILPE	**3.764E+02**	**7.085E+02**	**1.925E+02**	**5.900E+02**	2.074E+03
	ILPE	5.155E+02	1.109E+03	5.112E+02	9.752E+02	3.290E+03
	LPE	1.103E+03	8.494E+03	5.144E+03	6.718E+03	2.431E+04
	GPEA	1.458E+04	4.125E+04	2.197E+04	3.404E+04	1.014E+05
	GPEAae	7.642E+02	1.925E+03	6.054E+02	1.850E+03	3.037E+03
	GPEAed	1.777E+03	3.803E+03	1.676E+03	3.352E+03	9.319E+03
	NeGPEs	1.690E+03	3.144E+03	9.553E+02	2.964E+03	5.009E+03
	TOGPEAe	1.547E+03	4.619E+03	2.004E+03	4.499E+03	1.197E+04
	DE	9.619E+02	1.505E+03	2.282E+02	1.524E+03	**2.052E+03**
	ABC	1.382E+04	4.323E+04	1.309E+04	4.435E+04	6.597E+04
	GWO	4.067E+04	1.538E+05	1.032E+05	1.184E+05	4.406E+05

**Table 2:** Average ranking of TILPE and other considered algorithms for CEC2014 benchmark functions.

Statistic	TILPE	ILPE	LPE	GPEA	GPEAae	GPEAed	NeGPEs	TOGPEAe	DE	ABC	GWO
BEST	**1.9**	3.33	8.1	8	4.26	6.43	4.56	5.23	5.2	8.73	10.23
MEAN	**2.6**	3.2	8.13	7.73	5.4	7.43	4.37	5.93	3.6	7.67	9.93
STD	4.4	4.57	7.47	7.4	6.3	7.43	5.07	6.87	**1.6**	5.4	9.5
MED	**2.4**	3.2	8.2	7.67	5.77	7.5	4.2	5.6	3.63	7.93	9.9
MAX	2.87	3.63	8	7.9	5.63	7.9	4.37	6.57	**2.8**	6.53	9.8

### Comparison of TILPE with other state-of-the-art algorithms on CEC2017 benchmark functions

In this subsection the performance of the proposed TILPE is compared with the other considered state of the art algorithms (ILPE, LPE, GPEA, GPEAae, GPEAed, NeGPEs, TOGPEAe, DE, ABC, and GWO). The same control parameters and parameter settings as described before are followed. The comparison result among TILPE and other considered algorithms are shown in Table 3 including the same performance indicators BEST, MEAN, STD, MED, and MAX of the function error. In Table 4 average ranking of TILPE and other considered algorithms are presented. The best outcomes for each indication in evaluating the CEC2017 benchmark functions are displayed in bold. From the result in Table 4, it is seen that the performance of TILPE is better for BEST values with an average ranking 1.7 while the average ranking of the considered algorithms ILPE, LPE, GPEA, GPEAae, GPEAed, NeGPEs, TOGPEAe, DE, ABC, and GWO are 2.63, 5.53, 5.9, 10.57, 4.7, 10.43, 2.77, 5.93, 6.93, 8.9. Also, the average MEAN ranking of TILPE is 1.7 which is superior to other considered algorithms and the average STD ranking is 3.1 which is only inferior than DE but superior than other nine considered algorithms. Similarly, the average MED and MAX ranking are 1.77 and 1.87 which is superior than all considered algorithms. TILPE reaches better solution accuracy on functions $F_{2}$, $F_{5}$, $F_{7}$, $F_{8}$, $F_{11}$, $F_{13}$, $F_{15}$, $F_{17}-F_{20}$, $F_{22}-F_{25}$ and $F_{27}-F_{30}$. Also, TILPE has smallest MEAN values for the functions $F_{2}$, $F_{4}$, $F_{5}$, $F_{7}$, $F_{8}$, $F_{11}$, $F_{13}$, $F_{15}$, $F_{18}-F_{20}$, and $F_{22}-F_{30}$ and has smallest STD values for the functions $F_{2}$, $F_{13}$, $F_{18}$, $F_{22}$ and $F_{30}$. Moreover, TILPE gives better MED and MAX values for the functions $F_{2}$, $F_{4}$, $F_{5}$, $F_{7}$, $F_{8}$, $F_{11}-F_{13}$, $F_{15}$, $F_{18}-F_{20}$, $F_{23}-F_{30}$, and $F_{1}$, $F_{2}$, $F_{7}$, $F_{11}$, $F_{13}$, $F_{18}$, $F_{20}-F_{24}$, $F_{26}-F_{30}$. The outcome shows that TILPE performs both hybrid and composite functions quite well. It is evident from the above investigation that TILPE greatly outperforms its peers in terms of solution accuracy.

**Table 3:** Results of TILPE with other metaheuristic algorithms on CEC2017 benchmark functions

Function	Algorithm	BEST	MEAN	STD	MED	MAX
$F_{1}$	TILPE	1.083E+00	2.284E+03	3.378E+03	2.905E+02	**1.254E+04**
	ILPE	**4.742E-01**	**2.209E+03**	**3.184E+03**	**7.057E+01**	1.432E+04
	LPE	5.201E+09	1.165E+10	3.559E+09	1.098E+10	1.928E+10
	GPEA	2.092E+09	4.474E+09	1.505E+09	4.499E+09	8.663E+09
	GPEAae	3.344E+11	3.526E+11	8.124E+09	3.529E+11	3.736E+11
	GPEAed	4.139E-01	2.095E+06	2.743E+06	1.219E+03	1.902E+04
	NeGPEs	3.123E+11	3.529E+11	1.074E+10	3.538E+11	3.681E+11
	TOGPEAe	1.442E+01	2.763E+03	4.355E+03	5.639E+02	2.013E+04
	DE	9.521E+03	2.982E+04	1.151E+04	2.942E+04	5.808E+04
	ABC	3.832E+08	1.322E+09	5.004E+08	1.357E+09	2.640E+09
	GWO	4.753E+10	1.055E+11	2.970E+10	1.030E+11	1.748E+11
$F_{2}$	TILPE	**7.423E+02**	**2.886E+12**	**1.406E+13**	**7.991E+07**	**7.428E+13**
	ILPE	9.207E+04	1.065E+15	7.400E+15	3.958E+10	5.286E+16
	LPE	1.346E+20	1.895E+29	1.139E+30	3.931E+25	8.109E+30
	GPEA	1.112E+11	4.507E+16	2.483E+17	9.018E+14	1.768E+18
	GPEAae	7.551E+90	1.096E+93	1.165E+93	7.128E+92	6.986E+93
	GPEAed	1.174E+13	3.290E+19	2.300E+20	5.460E+16	2.555E+20
	NeGPEs	1.633E+91	9.194E+92	1.129E+93	6.026E+92	6.844E+93
	TOGPEAe	4.873E+10	1.653E+16	4.446E+16	1.489E+14	2.785E+17
	DE	2.240E+21	1.251E+25	4.723E+25	1.406E+24	3.329E+26
	ABC	1.593E+34	2.209E+34	5.306E+33	2.092E+34	4.610E+34
	GWO	1.573E+35	4.398E+42	1.622E+43	1.988E+39	8.155E+43
$F_{3}$	TILPE	9.758E+02	7.508E+03	4.485E+03	6.405E+03	1.703E+04
	ILPE	**2.025E+00**	**1.488E+02**	**2.602E+02**	**4.484E+01**	**1.057E+03**
	LPE	2.690E+04	4.663E+04	1.395E+04	4.580E+04	8.482E+04
	GPEA	1.810E+04	3.483E+04	6.979E+03	3.455E+04	4.882E+04
	GPEAae	4.616E+05	5.033E+05	2.079E+04	5.057E+05	5.374E+05
	GPEAed	1.927E+02	1.267E+04	5.627E+03	2.908E+03	1.079E+04
	NeGPEs	4.403E+05	5.093E+05	2.294E+04	5.133E+05	5.479E+05
	TOGPEAe	2.020E+02	2.311E+03	1.539E+03	2.063E+03	6.432E+03
	DE	3.404E+04	5.589E+04	7.859E+03	5.690E+04	6.977E+04
	ABC	5.284E+04	9.058E+04	1.065E+04	9.327E+04	1.049E+05
	GWO	1.883E+05	3.263E+05	5.912E+04	3.351E+05	4.255E+05
$F_{4}$	TILPE	1.526E-02	**5.813E+01**	4.027E+01	**7.204E+01**	1.200E+02
	ILPE	**1.192E-02**	6.574E+01	3.028E+01	7.238E+01	**1.134E+02**
	LPE	3.166E+02	1.414E+03	7.202E+02	1.261E+03	3.897E+03
	GPEA	4.810E+02	8.956E+02	2.642E+02	8.647E+02	1.759E+03
	GPEAae	1.891E+05	2.323E+05	1.300E+04	2.346E+05	2.608E+05
	GPEAed	5.944E-01	1.227E+02	2.741E+01	8.562E+01	1.265E+02
	NeGPEs	2.078E+05	2.348E+05	1.018E+04	2.352E+05	2.536E+05
	TOGPEAe	1.283E+01	9.780E+01	3.676E+01	8.978E+01	1.981E+02
	DE	8.574E+01	8.878E+01	**4.759E+00**	8.787E+01	1.186E+02
	ABC	2.333E+02	3.460E+02	6.227E+01	3.343E+02	5.151E+02
	GWO	3.209E+04	9.531E+04	4.135E+04	8.555E+04	2.356E+05
$F_{5}$	TILPE	**1.990E+01**	**4.781E+01**	2.063E+01	**4.378E+01**	1.273E+02
	ILPE	3.880E+01	7.144E+01	1.773E+01	6.865E+01	**1.252E+02**
	LPE	1.002E+02	1.607E+02	2.613E+01	1.561E+02	2.112E+02
	GPEA	6.559E+01	9.805E+01	1.684E+01	9.785E+01	1.351E+02
	GPEAae	1.443E+03	1.523E+03	3.540E+01	1.523E+03	1.589E+03
	GPEAed	5.572E+01	1.122E+02	2.391E+01	1.194E+02	3.211E+02
	NeGPEs	1.423E+03	1.529E+03	3.095E+01	1.529E+03	1.595E+03
	TOGPEAe	3.383E+01	5.958E+01	1.876E+01	5.771E+01	1.264E+02
	DE	1.751E+02	2.097E+02	**1.285E+01**	2.124E+02	2.332E+02
	ABC	1.868E+02	2.216E+02	1.662E+01	2.238E+02	2.511E+02
	GWO	4.281E+02	6.611E+02	9.799E+01	6.597E+02	9.659E+02
$F_{6}$	TILPE	7.349E-02	1.645E+00	1.221E+00	1.261E+00	6.643E+00
	ILPE	1.065E+01	1.989E+01	4.463E+00	2.040E+01	2.849E+01
	LPE	1.628E+01	2.351E+01	4.067E+00	2.371E+01	3.359E+01
	GPEA	1.476E+01	2.213E+01	3.752E+00	2.217E+01	2.971E+01
	GPEAae	1.406E+02	1.506E+02	3.694E+00	1.514E+02	1.562E+02
	GPEAed	1.426E+01	2.690E+01	6.263E+00	3.505E+01	6.810E+01
	NeGPEs	1.339E+02	1.498E+02	4.084E+00	1.505E+02	1.556E+02
	TOGPEAe	5.185E+00	1.134E+01	2.775E+00	1.144E+01	1.845E+01
	DE	**1.669E-02**	**3.156E-02**	**1.105E-02**	**2.823E-02**	**6.071E-02**
	ABC	1.926E+01	2.751E+01	3.338E+00	2.785E+01	3.311E+01
	GWO	1.167E+02	1.481E+02	1.401E+01	1.492E+02	1.727E+02
$F_{7}$	TILPE	**5.359E+01**	**7.394E+01**	1.534E+01	**7.045E+01**	**1.205E+02**
	ILPE	8.150E+01	1.544E+02	2.995E+01	1.532E+02	2.192E+02
	LPE	1.891E+02	2.749E+02	4.141E+01	2.730E+02	3.576E+02
	GPEA	1.318E+02	2.264E+02	4.286E+01	2.237E+02	3.351E+02
	GPEAae	6.807E+03	7.338E+03	1.687E+02	7.328E+03	7.631E+03
	GPEAed	2.002E+02	2.662E+02	5.338E+01	3.222E+02	5.953E+02
	NeGPEs	6.822E+03	7.331E+03	1.888E+02	7.381E+03	7.606E+03
	TOGPEAe	1.250E+02	2.110E+02	5.670E+01	2.056E+02	3.617E+02
	DE	2.252E+02	2.518E+02	**1.261E+01**	2.536E+02	2.771E+02
	ABC	2.989E+02	3.721E+02	3.250E+01	3.757E+02	4.281E+02
	GWO	1.673E+03	2.502E+03	4.749E+02	2.504E+03	3.532E+03
$F_{8}$	TILPE	**2.288E+01**	**5.763E+01**	2.273E+01	**4.975E+01**	1.229E+02
	ILPE	2.587E+01	6.129E+01	2.111E+01	5.870E+01	1.353E+02
	LPE	9.779E+01	1.497E+02	2.124E+01	1.520E+02	2.070E+02
	GPEA	5.148E+01	8.705E+01	1.498E+01	8.840E+01	**1.216E+02**
	GPEAae	1.760E+03	1.859E+03	3.785E+01	1.862E+03	1.914E+03
	GPEAed	4.676E+01	8.237E+01	1.719E+01	9.452E+01	2.831E+02
	NeGPEs	1.771E+03	1.869E+03	3.442E+01	1.869E+03	1.927E+03
	TOGPEAe	2.487E+01	5.831E+01	2.328E+01	5.273E+01	1.689E+02
	DE	1.756E+02	2.107E+02	**1.294E+01**	2.136E+02	2.366E+02
	ABC	1.732E+02	2.154E+02	1.639E+01	2.188E+02	2.519E+02
	GWO	3.322E+02	4.966E+02	6.133E+01	5.037E+02	6.261E+02
$F_{9}$	TILPE	9.270E+00	7.920E+01	4.499E+01	7.233E+01	2.305E+02
	ILPE	3.886E+02	8.900E+02	3.074E+02	8.470E+02	1.738E+03
	LPE	5.555E+02	1.726E+03	6.515E+02	1.687E+03	3.331E+03
	GPEA	7.660E+02	1.252E+03	2.362E+02	1.207E+03	1.762E+03
	GPEAae	7.077E+04	9.194E+04	6.334E+03	9.241E+04	1.036E+05
	GPEAed	1.051E+03	1.418E+03	4.457E+02	1.982E+03	7.663E+03
	NeGPEs	8.347E+04	9.488E+04	5.365E+03	9.555E+04	1.064E+05
	TOGPEAe	1.870E+02	5.593E+02	2.527E+02	5.007E+02	1.849E+03
	DE	**1.842E-02**	**4.151E-01**	**4.084E-01**	**3.395E-01**	**1.927E+00**
	ABC	2.004E+03	4.378E+03	8.633E+02	4.379E+03	6.172E+03
	GWO	2.073E+04	3.151E+04	5.409E+03	3.105E+04	4.601E+04
$F_{10}$	TILPE	2.564E+03	5.774E+03	1.267E+03	6.104E+03	7.335E+03
	ILPE	2.249E+03	3.964E+03	5.775E+02	4.032E+03	5.018E+03
	LPE	6.054E+03	7.165E+03	4.821E+02	7.207E+03	8.207E+03
	GPEA	**1.884E+03**	**3.637E+03**	1.368E+03	**3.072E+03**	7.462E+03
	GPEAae	1.451E+04	1.526E+04	3.621E+02	1.532E+04	1.594E+04
	GPEAed	5.925E+03	7.075E+03	1.555E+03	8.100E+03	8.731E+03
	NeGPEs	1.448E+04	1.523E+04	2.973E+02	1.528E+04	1.573E+04
	TOGPEAe	2.053E+03	3.682E+03	9.396E+02	3.452E+03	7.361E+03
	DE	6.384E+03	7.103E+03	**2.464E+02**	7.141E+03	7.475E+03
	ABC	3.743E+03	4.460E+03	2.668E+02	4.464E+03	**4.892E+03**
	GWO	2.849E+03	4.617E+03	6.084E+02	4.634E+03	5.915E+03
$F_{11}$	TILPE	**4.060E+00**	**2.185E+01**	2.215E+01	**1.417E+01**	**8.593E+01**
	ILPE	2.291E+01	6.156E+01	2.407E+01	5.399E+01	1.217E+02
	LPE	1.755E+02	3.605E+02	1.565E+02	3.150E+02	8.449E+02
	GPEA	1.914E+02	3.941E+02	1.224E+02	3.723E+02	6.780E+02
	GPEAae	7.426E+04	8.469E+04	4.655E+03	8.426E+04	9.530E+04
	GPEAed	3.145E+01	9.771E+01	4.029E+01	1.020E+02	4.160E+02
	NeGPEs	6.199E+04	8.444E+04	5.017E+03	8.507E+04	9.213E+04
	TOGPEAe	1.198E+01	6.973E+01	4.189E+01	5.978E+01	1.792E+02
	DE	8.177E+01	1.105E+02	**1.994E+01**	1.070E+02	1.621E+02
	ABC	8.201E+02	1.349E+03	3.467E+02	1.317E+03	2.089E+03
	GWO	6.155E+03	1.933E+04	8.763E+03	1.653E+04	4.292E+04
$F_{12}$	TILPE	2.361E+03	3.359E+04	6.233E+04	**1.703E+04**	3.817E+05
	ILPE	**1.342E+03**	3.409E+04	3.824E+04	2.867E+04	2.416E+05
	LPE	9.399E+06	3.751E+08	3.460E+08	3.013E+08	1.767E+09
	GPEA	1.623E+07	2.684E+08	1.415E+08	2.583E+08	6.056E+08
	GPEAae	1.984E+11	2.169E+11	5.557E+09	2.185E+11	2.266E+11
	GPEAed	1.966E+03	3.052E+05	7.630E+05	2.771E+04	5.106E+05
	NeGPEs	1.989E+11	2.159E+11	5.120E+09	2.163E+11	2.273E+11
	TOGPEAe	2.561E+03	**2.521E+04**	**2.482E+04**	1.722E+04	**1.416E+05**
	DE	3.671E+06	9.388E+06	4.218E+06	8.222E+06	2.002E+07
	ABC	6.624E+07	1.923E+08	6.806E+07	1.822E+08	3.927E+08
	GWO	1.719E+10	2.782E+10	8.343E+09	2.638E+10	4.968E+10
$F_{13}$	TILPE	**2.784E+01**	**1.878E+02**	**1.277E+02**	**1.395E+02**	**5.318E+02**
	ILPE	1.585E+02	8.346E+02	5.028E+02	6.794E+02	2.383E+03
	LPE	1.886E+03	1.389E+04	1.191E+04	9.904E+03	5.391E+04
	GPEA	9.140E+02	3.072E+06	1.396E+07	1.093E+04	8.260E+07
	GPEAae	1.288E+11	1.389E+11	3.956E+09	1.395E+11	1.493E+11
	GPEAed	8.337E+02	2.077E+04	1.804E+04	1.261E+04	6.127E+04
	NeGPEs	1.272E+11	1.381E+11	5.097E+09	1.388E+11	1.503E+11
	TOGPEAe	1.112E+02	1.592E+04	1.565E+04	1.119E+04	5.150E+04
	DE	2.293E+03	3.914E+03	8.820E+02	3.807E+03	5.728E+03
	ABC	3.744E+07	1.208E+08	5.184E+07	1.167E+08	2.914E+08
	GWO	3.026E+10	3.443E+10	7.131E+09	3.026E+10	5.497E+10
$F_{14}$	TILPE	3.990E+01	7.774E+01	1.581E+01	7.781E+01	1.012E+02
	ILPE	2.944E+01	5.747E+01	1.659E+01	5.494E+01	1.045E+02
	LPE	3.747E+01	5.939E+01	1.284E+01	5.979E+01	**9.138E+01**
	GPEA	1.299E+02	6.576E+02	6.244E+02	4.279E+02	3.390E+03
	GPEAae	5.887E+08	9.338E+08	1.328E+08	9.305E+08	1.235E+09
	GPEAed	5.208E+01	1.157E+02	1.035E+02	1.246E+02	1.158E+03
	NeGPEs	6.404E+08	9.205E+08	1.229E+08	9.324E+08	1.166E+09
	TOGPEAe	**2.541E+01**	**5.014E+01**	2.302E+01	**4.430E+01**	1.373E+02
	DE	7.545E+01	9.175E+01	**6.906E+00**	9.288E+01	1.061E+02
	ABC	3.590E+04	3.399E+05	1.693E+05	3.232E+05	9.072E+05
	GWO	5.422E+06	9.857E+06	8.750E+06	6.409E+06	4.266E+07
$F_{15}$	TILPE	**8.885E+00**	**9.563E+01**	6.501E+01	**1.029E+02**	3.461E+02
	ILPE	3.318E+01	1.764E+02	8.081E+01	1.674E+02	3.542E+02
	LPE	1.311E+02	6.513E+02	3.481E+02	5.855E+02	1.373E+03
	GPEA	5.762E+02	3.729E+03	1.812E+03	3.292E+03	9.905E+03
	GPEAae	6.289E+10	6.893E+10	3.096E+09	6.901E+10	7.540E+10
	GPEAed	4.193E+01	2.661E+03	3.795E+03	2.805E+02	3.611E+04
	NeGPEs	6.139E+10	6.875E+10	3.295E+09	6.894E+10	7.566E+10
	TOGPEAe	5.841E+01	6.195E+02	7.410E+02	2.562E+02	3.540E+03
	DE	1.003E+02	1.454E+02	**2.192E+01**	1.429E+02	**2.080E+02**
	ABC	1.893E+06	1.409E+07	7.604E+06	1.318E+07	3.408E+07
	GWO	6.181E+09	6.181E+09	1.329E+05	6.181E+09	6.181E+09
$F_{16}$	TILPE	3.661E+02	1.319E+03	2.618E+02	1.385E+03	1.788E+03
	ILPE	3.863E+02	**8.519E+02**	2.872E+02	**8.736E+02**	1.731E+03
	LPE	3.355E+02	1.071E+03	4.578E+02	1.090E+03	1.961E+03
	GPEA	**2.248E+02**	9.227E+02	3.264E+02	8.925E+02	**1.670E+03**
	GPEAae	3.033E+04	3.389E+04	1.721E+03	3.405E+04	3.749E+04
	GPEAed	5.164E+02	1.033E+03	3.947E+02	1.318E+03	2.933E+03
	NeGPEs	2.911E+04	3.405E+04	1.853E+03	3.410E+04	3.794E+04
	TOGPEAe	2.569E+02	1.124E+03	4.418E+02	1.111E+03	2.015E+03
	DE	1.064E+03	1.552E+03	1.693E+02	1.581E+03	1.775E+03
	ABC	8.393E+02	1.368E+03	**1.663E+02**	1.381E+03	1.687E+03
	GWO	1.518E+03	2.356E+03	3.877E+02	2.349E+03	3.171E+03
$F_{17}$	TILPE	**1.182E+01**	2.229E+02	1.604E+02	2.457E+02	6.061E+02
	ILPE	5.722E+01	3.028E+02	2.109E+02	3.026E+02	7.434E+02
	LPE	5.815E+01	**2.031E+02**	1.843E+02	**1.241E+02**	8.055E+02
	GPEA	7.135E+01	2.687E+02	1.398E+02	3.200E+02	6.651E+02
	GPEAae	7.169E+06	1.324E+07	2.966E+06	1.299E+07	1.865E+07
	GPEAed	7.917E+01	4.997E+02	2.379E+02	6.313E+02	1.488E+03
	NeGPEs	7.292E+06	1.350E+07	2.741E+06	1.388E+07	1.773E+07
	TOGPEAe	6.211E+01	4.544E+02	2.309E+02	4.139E+02	9.551E+02
	DE	8.712E+01	3.442E+02	1.178E+02	3.597E+02	v**5.443E+02**
	ABC	3.207E+02	5.535E+02	**8.460E+01**	5.588E+02	7.230E+02
	GWO	5.156E+03	5.795E+03	3.948E+02	5.755E+03	6.673E+03
$F_{18}$	TILPE	**5.093E+01**	**8.568E+01**	**1.691E+01**	**8.529E+01**	**1.322E+02**
	ILPE	5.542E+01	9.809E+01	2.846E+01	9.301E+01	1.879E+02
	LPE	3.173E+02	1.168E+04	1.279E+04	7.548E+03	7.038E+04
	GPEA	1.419E+04	6.049E+04	3.707E+04	5.043E+04	1.654E+05
	GPEAae	1.444E+09	1.941E+09	2.312E+08	1.967E+09	2.363E+09
	GPEAed	2.636E+02	9.403E+03	8.029E+03	5.452E+03	3.736E+04
	NeGPEs	1.373E+09	1.844E+09	2.364E+08	1.870E+09	2.339E+09
	TOGPEAe	2.196E+02	4.350E+03	3.322E+03	3.319E+03	1.346E+04
	DE	2.482E+04	6.298E+04	2.572E+04	6.192E+04	1.491E+05
	ABC	2.279E+05	9.112E+05	3.238E+05	8.821E+05	1.708E+06
	GWO	2.486E+05	3.660E+06	7.444E+06	1.028E+06	5.071E+07
$F_{19}$	TILPE	**7.032E+00**	**4.835E+01**	2.775E+01	**4.562E+01**	1.231E+02
	ILPE	2.604E+01	7.426E+01	2.752E+01	7.055E+01	1.582E+02
	LPE	1.628E+01	1.005E+03	1.785E+03	1.875E+02	7.726E+03
	GPEA	6.571E+01	2.620E+03	2.353E+03	2.030E+03	1.043E+04
	GPEAae	1.076E+10	1.255E+10	8.189E+08	1.273E+10	1.389E+10
	GPEAed	5.493E+01	2.966E+03	4.612E+03	1.588E+03	3.421E+04
	NeGPEs	1.110E+10	1.275E+10	7.813E+08	1.273E+10	1.429E+10
	TOGPEAe	1.672E+01	9.157E+03	1.019E+04	5.463E+03	4.140E+04
	DE	3.632E+01	4.862E+01	**5.464E+00**	4.920E+01	**5.881E+01**
	ABC	1.664E+06	1.644E+07	7.780E+06	1.580E+07	3.123E+07
	GWO	6.646E+09	6.692E+09	1.047E+08	6.661E+09	7.129E+09
$F_{20}$	TILPE	**4.627E+00**	**2.254E+02**	1.266E+02	**1.991E+02**	**4.864E+02**
	ILPE	1.712E+02	4.621E+02	1.583E+02	4.740E+02	7.659E+02
	LPE	5.212E+01	2.798E+02	2.077E+02	2.383E+02	9.217E+02
	GPEA	9.943E+01	3.620E+02	1.344E+02	3.433E+02	7.134E+02
	GPEAae	3.011E+03	3.376E+03	1.658E+02	3.388E+03	3.727E+03
	GPEAed	5.141E+02	5.945E+02	2.443E+02	1.149E+03	1.416E+03
	NeGPEs	2.933E+03	3.410E+03	1.536E+02	3.419E+03	3.674E+03
	TOGPEAe	9.623E+01	4.912E+02	2.104E+02	4.914E+02	9.351E+02
	DE	5.517E+01	2.772E+02	1.885E+02	2.582E+02	6.195E+02
	ABC	2.133E+02	5.425E+02	**1.049E+02**	5.528E+02	7.527E+02
	GWO	7.855E+02	1.047E+03	1.315E+02	1.053E+03	1.452E+03
$F_{21}$	TILPE	2.105E+02	2.421E+02	2.008E+01	2.371E+02	**2.950E+02**
	ILPE	2.414E+02	2.657E+02	1.615E+01	2.647E+02	3.176E+02
	LPE	2.930E+02	3.437E+02	2.237E+01	3.444E+02	3.952E+02
	GPEA	2.626E+02	3.005E+02	1.618E+01	2.992E+02	3.322E+02
	GPEAae	1.552E+03	1.619E+03	2.699E+01	1.627E+03	1.665E+03
	GPEAed	2.411E+02	2.991E+02	2.024E+01	3.034E+02	5.277E+02
	NeGPEs	1.554E+03	1.620E+03	2.761E+01	1.624E+03	1.674E+03
	TOGPEAe	2.276E+02	2.622E+02	1.979E+01	2.628E+02	3.200E+02
	DE	3.867E+02	4.064E+02	**1.024E+01**	4.065E+02	4.277E+02
	ABC	**1.643E+02**	**2.365E+02**	4.736E+01	**2.262E+02**	3.934E+02
	GWO	7.062E+02	8.420E+02	7.364E+01	8.359E+02	1.072E+03
$F_{22}$	TILPE	**1.000E+02**	**1.000E+02**	**7.049E-07**	1.000E+02	**1.000E+02**
	ILPE	1.000E+02	1.000E+02	7.056E-07	**1.000E+02**	1.000E+02
	LPE	6.961E+02	1.213E+03	3.251E+02	1.191E+03	2.019E+03
	GPEA	3.862E+02	7.675E+02	2.602E+02	6.879E+02	1.786E+03
	GPEAae	1.352E+04	1.510E+04	4.818E+02	1.520E+04	1.583E+04
	GPEAed	1.000E+02	2.474E+03	3.456E+03	1.048E+02	9.242E+03
	NeGPEs	1.337E+04	1.493E+04	4.732E+02	1.505E+04	1.564E+04
	TOGPEAe	1.000E+02	3.719E+03	1.696E+03	4.000E+03	7.451E+03
	DE	1.000E+02	1.000E+02	1.797E-05	1.000E+02	1.000E+02
	ABC	1.894E+02	2.604E+02	3.745E+01	2.574E+02	3.404E+02
	GWO	3.757E+03	4.740E+03	5.679E+02	4.675E+03	5.916E+03
$F_{23}$	TILPE	**3.635E+02**	**3.914E+02**	1.598E+01	**3.892E+02**	**4.426E+02**
	ILPE	4.034E+02	4.801E+02	3.266E+01	4.757E+02	5.499E+02
	LPE	4.958E+02	5.594E+02	3.672E+01	5.517E+02	6.552E+02
	GPEA	4.695E+02	5.508E+02	3.867E+01	5.474E+02	6.652E+02
	GPEAae	4.750E+03	5.067E+03	1.326E+02	5.088E+03	5.335E+03
	GPEAed	4.204E+02	4.915E+02	3.747E+01	4.973E+02	7.054E+02
	NeGPEs	4.631E+03	5.047E+03	1.660E+02	5.084E+03	5.294E+03
	TOGPEAe	3.736E+02	4.360E+02	2.856E+01	4.309E+02	5.186E+02
	DE	5.287E+02	5.601E+02	**1.067E+01**	5.605E+02	5.768E+02
	ABC	4.372E+02	5.228E+02	6.116E+01	5.074E+02	6.537E+02
	GWO	8.149E+02	9.650E+02	8.987E+01	9.634E+02	1.196E+03
$F_{24}$	TILPE	**4.380E+02**	4**.610E+02**	1.593E+01	**4.609E+02**	**5.103E+02**
	ILPE	4.844E+02	5.629E+02	3.577E+01	5.636E+02	6.446E+02
	LPE	5.545E+02	6.370E+02	3.786E+01	6.378E+02	7.130E+02
	GPEA	5.745E+02	6.498E+02	4.083E+01	6.515E+02	7.290E+02
	GPEAae	4.445E+03	4.696E+03	1.086E+02	4.722E+03	4.922E+03
	GPEAed	5.149E+02	5.886E+02	4.013E+01	5.743E+02	7.094E+02
	NeGPEs	4.450E+03	4.706E+03	1.120E+02	4.719E+03	4.886E+03
	TOGPEAe	4.750E+02	5.182E+02	2.718E+01	5.107E+02	6.017E+02
	DE	5.987E+02	6.258E+02	**9.044E+00**	6.260E+02	6.441E+02
	ABC	4.910E+02	7.366E+02	7.958E+01	7.651E+02	8.220E+02
	GWO	8.849E+02	1.045E+03	7.226E+01	1.024E+03	1.213E+03
$F_{25}$	TILPE	**3.834E+02**	**3.864E+02**	1.556E+00	**3.868E+02**	3.898E+02
	ILPE	3.834E+02	3.876E+02	2.858E+00	3.868E+02	4.008E+02
	LPE	5.337E+02	8.429E+02	1.697E+02	8.309E+02	1.345E+03
	GPEA	5.370E+02	6.394E+02	5.501E+01	6.381E+02	7.682E+02
	GPEAae	5.575E+04	6.431E+04	3.544E+03	6.533E+04	7.049E+04
	GPEAed	3.835E+02	4.139E+02	2.105E+01	3.922E+02	4.590E+02
	NeGPEs	5.410E+04	6.386E+04	3.067E+03	6.386E+04	6.872E+04
	TOGPEAe	3.844E+02	3.983E+02	1.594E+01	3.890E+02	4.373E+02
	DE	3.870E+02	3.871E+02	**4.021E-02**	3.871E+02	**3.872E+02**
	ABC	4.950E+02	6.098E+02	4.495E+01	6.130E+02	6.943E+02
	GWO	6.943E+03	1.689E+04	5.688E+03	1.694E+04	3.008E+04
$F_{26}$	TILPE	2.000E+02	**1.164E+03**	4.878E+02	**1.316E+03**	**1.870E+03**
	ILPE	**2.000E+02**	1.919E+03	7.952E+02	2.165E+03	3.092E+03
	LPE	1.597E+03	3.291E+03	5.349E+02	3.348E+03	4.591E+03
	GPEA	1.573E+03	3.636E+03	9.509E+02	3.895E+03	4.868E+03
	GPEAae	7.629E+04	8.812E+04	3.578E+03	8.860E+04	9.263E+04
	GPEAed	1.900E+03	2.626E+03	3.573E+02	2.501E+03	3.746E+03
	NeGPEs	7.575E+04	8.744E+04	3.306E+03	8.834E+04	9.217E+04
	TOGPEAe	1.435E+03	1.975E+03	2.319E+02	1.982E+03	2.508E+03
	DE	2.778E+03	3.047E+03	**1.398E+02**	3.088E+03	3.289E+03
	ABC	1.377E+03	2.028E+03	3.459E+02	2.110E+03	2.703E+03
	GWO	4.457E+03	7.189E+03	9.381E+02	7.194E+03	9.192E+03
$F_{27}$	TILPE	**4.781E+02**	**5.177E+02**	1.561E+01	**5.171E+02**	**5.475E+02**
	ILPE	4.995E+02	5.545E+02	2.898E+01	5.497E+02	6.378E+02
	LPE	5.242E+02	6.056E+02	3.415E+01	6.011E+02	6.741E+02
	GPEA	5.870E+02	6.641E+02	3.199E+01	6.568E+02	7.233E+02
	GPEAae	2.249E+04	2.461E+04	9.902E+02	2.452E+04	2.659E+04
	GPEAed	5.222E+02	5.881E+02	2.647E+01	5.778E+02	7.093E+02
	NeGPEs	2.218E+04	2.477E+04	9.306E+02	2.488E+04	2.639E+04
	TOGPEAe	5.243E+02	5.508E+02	1.625E+01	5.484E+02	6.054E+02
	DE	5.397E+02	5.621E+02	**9.899E+00**	5.612E+02	5.808E+02
	ABC	5.423E+02	5.830E+02	1.527E+01	5.839E+02	6.172E+02
	GWO	9.384E+02	1.265E+03	2.323E+02	1.220E+03	1.818E+03
$F_{28}$	TILPE	**3.000E+02**	**3.468E+02**	5.579E+01	**3.005E+02**	**4.546E+02**
	ILPE	3.000E+02	3.609E+02	5.972E+01	3.908E+02	4.602E+02
	LPE	5.753E+02	1.014E+03	2.172E+02	9.710E+02	1.693E+03
	GPEA	6.557E+02	8.509E+02	8.965E+01	8.548E+02	1.076E+03
	GPEAae	2.928E+04	3.117E+04	8.190E+02	3.117E+04	3.264E+04
	GPEAed	3.995E+02	4.618E+02	3.213E+01	4.254E+02	4.938E+02
	NeGPEs	2.688E+04	3.090E+04	1.160E+03	3.122E+04	3.283E+04
	TOGPEAe	3.385E+02	4.281E+02	2.695E+01	4.221E+02	5.064E+02
	DE	3.526E+02	4.280E+02	**2.116E+01**	4.244E+02	4.802E+02
	ABC	6.371E+02	8.760E+02	1.076E+02	8.755E+02	1.066E+03
	GWO	8.160E+03	1.497E+04	3.983E+03	1.487E+04	2.630E+04
$F_{29}$	TILPE	**4.144E+02**	**5.422E+02**	1.464E+02	**4.854E+02**	**1.074E+03**
	ILPE	4.596E+02	6.680E+02	2.071E+02	5.851E+02	1.355E+03
	LPE	5.289E+02	7.471E+02	1.380E+02	7.089E+02	1.196E+03
	GPEA	6.464E+02	9.906E+02	2.151E+02	9.663E+02	1.598E+03
	GPEAae	3.216E+06	9.971E+06	2.415E+06	9.935E+06	1.642E+07
	GPEAed	5.861E+02	8.951E+02	2.071E+02	1.021E+03	1.710E+03
	NeGPEs	5.292E+06	1.046E+07	2.390E+06	1.049E+07	1.520E+07
	TOGPEAe	4.554E+02	8.997E+02	2.438E+02	8.924E+02	1.502E+03
	DE	1.036E+03	1.291E+03	1.341E+02	1.298E+03	1.525E+03
	ABC	1.098E+03	1.297E+03	**1.200E+02**	1.262E+03	1.576E+03
	GWO	1.155E+03	1.681E+03	2.995E+02	1.687E+03	2.366E+03
$F_{30}$	TILPE	**2.107E+03**	**4.771E+03**	**2.648E+03**	**3.791E+03**	**1.016E+04**
	ILPE	2.233E+03	4.998E+03	2.653E+03	4.121E+03	1.347E+04
	LPE	1.338E+04	1.282E+05	1.450E+05	7.095E+04	6.036E+05
	GPEA	7.042E+04	7.757E+05	7.494E+05	5.301E+05	2.945E+06
	GPEAae	1.822E+10	2.076E+10	9.999E+08	2.081E+10	2.287E+10
	GPEAed	2.463E+03	8.107E+03	4.622E+03	6.269E+03	1.503E+04
	NeGPEs	1.843E+10	2.076E+10	1.029E+09	2.081E+10	2.255E+10
	TOGPEAe	2.793E+03	7.348E+03	3.010E+03	7.242E+03	1.286E+04
	DE	6.597E+04	1.717E+05	6.459E+04	1.593E+05	3.919E+05
	ABC	1.681E+06	7.759E+06	3.746E+06	7.477E+06	1.607E+07
	GWO	2.634E+09	4.568E+09	8.608E+08	4.686E+09	6.043E+09

**Table 4:** Average ranking of TILPE and other considered algorithms for CEC2017 benchmark functions.

Statistic	TILPE	ILPE	LPE	GPEA	GPEAae	GPEAed	NeGPEs	TOGPEAe	DE	ABC	GWO
BEST	**1.7**	2.63	5.53	5.9	10.57	4.7	10.43	2.77	5.93	6.93	8.9
MEAN	**1.7**	2.6	5.77	5.5	10.5	5.53	10.5	3.5	4.87	6.7	8.83
STD	3.1	3.7	6.07	5.73	9.36	6.57	9.3	4.83	**2.36**	5.5	9.47
MED	**1.77**	2.6	5.8	5.53	10.47	5.3	10.53	3.47	5	6.7	8.83
MAX	**1.87**	2.7	5.97	5.53	10.6	6.33	10.33	4.1	3.7	6.07	8.8

### Convergence analysis

**Fig. 1:**
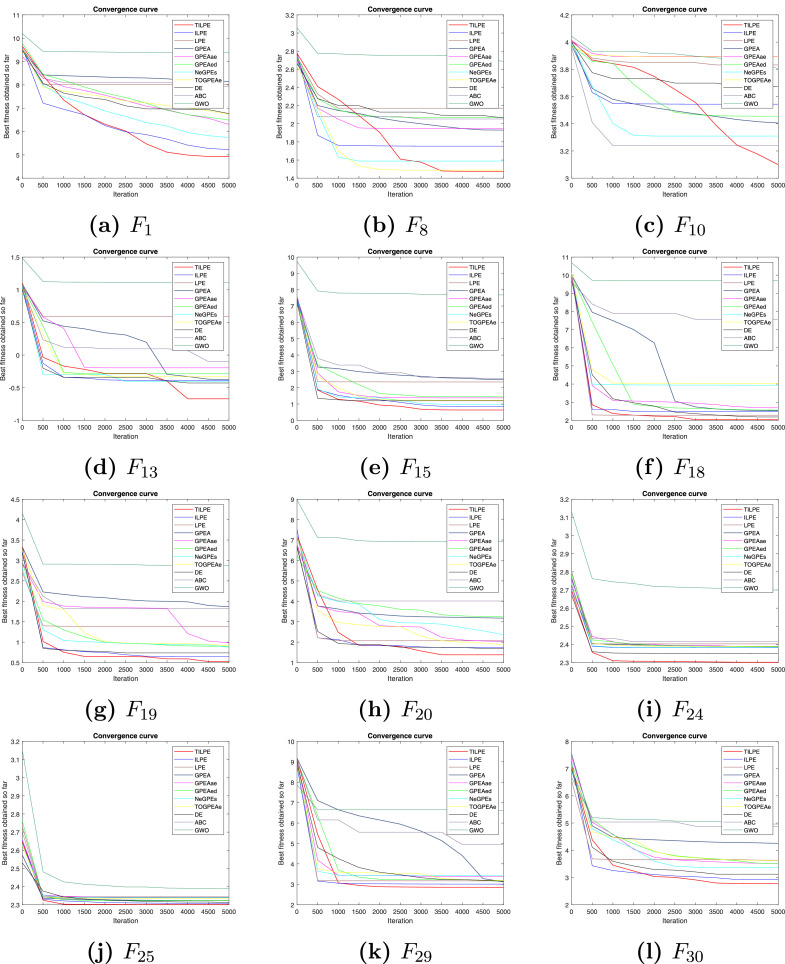
Convergence curves for CEC2014 benchmark functions

**Fig. 2:**
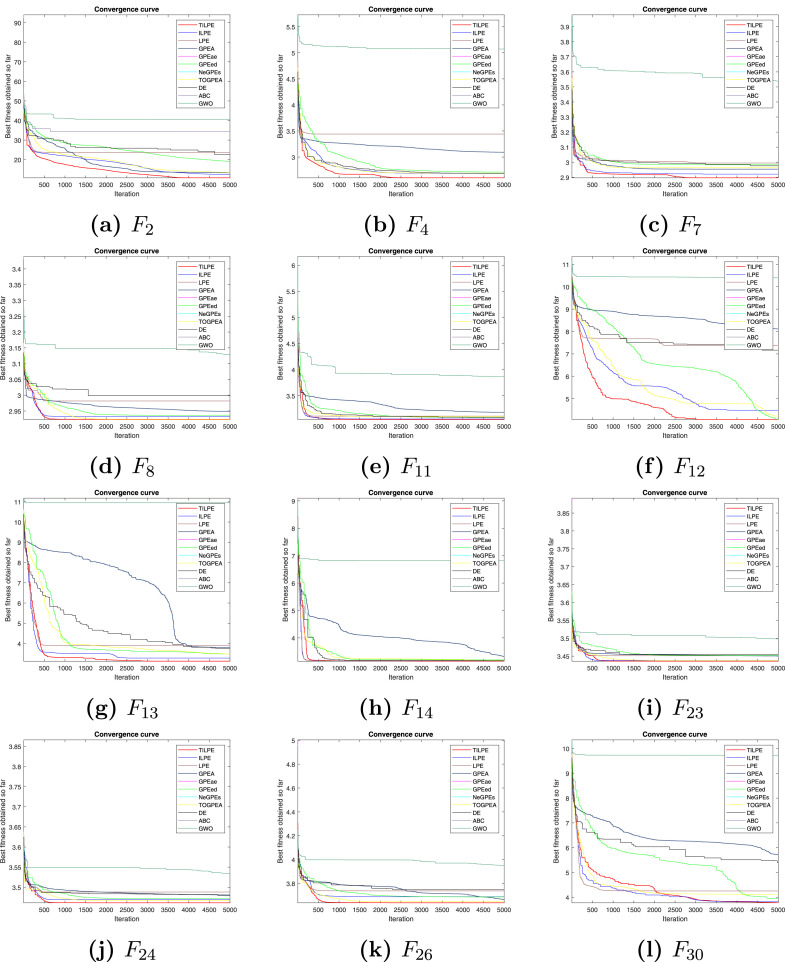
Convergence curves for CEC2017 benchmark functions

The convergence rate in the proposed TILPE is compared through convergence curves for selected well-known benchmark problems from CEC2014 and CEC2017 benchmark functions. It is shown in Figs. 1 and 2. The horizontal axis in both figures represents the maximum number of iterations, while the vertical axis indicates the logarithmic best value of the objective function. To demonstrate the convergence curves for the CEC2014 benchmark functions, we have selected the functions $F_{1}$, $F_{8}$, $F_{10}$, $F_{13}$, $F_{15}$, $F_{18}$, $F_{19}$, $F_{20}$, $F_{24}$, $F_{25}$, $F_{29}$, and $F_{30}$. Also, the functions $F_{2}$, $F_{4}$, $F_{7}$, $F_{8}$, $F_{11}$, $F_{12}$, $F_{13}$, $F_{14}$, $F_{23}$, $F_{24}$, $F_{26}$, and $F_{30}$ is selected for CEC2017 benchmark functions to show its convergence rate. These figures demonstrate that TILPE achieves not only a faster convergence rate but also a higher level of accuracy compared to its competitors. Consequently, based on the aforementioned findings, it can be concluded that TILPE exhibits superior overall performance compared to the other ten competing algorithms for the CEC2014 and CEC2017 benchmark functions.

### Statistical analysis

This section employs the Wilcoxon signed-rank test [40], [41] to examine the significant differences between TILPE and its competing algorithms. This non-parametric test determines if the distinction between TILPE and other metaheuristic algorithms is significant enough. To compare TILPE with other considered algorithms, this paper employs the optimal values (BEST) from Tables 1 and 3. This test is conducted in pairs with a significance level of 5% using the null hypothesis. The null hypothesis is rejected if the p-value is less than 0.05. The test results are represented as ‘+’, ‘≈’, and ‘-’ when TILPE performs better, comparably, or inferiorly to other considered algorithms, respectively. Out of 300 comparisons, 253 and 260 show positive signs for CEC2014 and CEC2017 benchmark functions respectively, and the results are presented in Tables 5 and 6, along with the corresponding p-values. Based on the analyses, TILPE performs remarkably better in terms of accuracy, robustness, and reliability than the other algorithms. It represents a significant improvement of TILPE over the proposed algorithm.

**Table 5:** Wilcoxon Signed-Rank Test Results for CEC2014 Benchmark Problems (BP); p-value denoted as p-val)

BP		ILPE	LPE	GPEA	GPEae	GPEed	NeGPEs	TOGPEAe	DE	ABC	GWO
$F_{1}$	p-val	1	3.304E-18	3.304E-18	8.441E-18	3.304E-18	2.572E-16	1.556E-16	3.304E-18	3.304E-18	3.304E-18
	outcome	≈	+	+	+	+	+	+	+	+	+
$F_{2}$	p-val	1	3.304E-18	3.304E-18	7.963E-18	3.304E-18	1	4.608E-15	2.679E-19	3.304E-18	3.304E-18
	outcome	≈	+	+	+	+	≈	+	-	+	+
$F_{3}$	p-val	1	3.304E-18	3.304E-18	4.987E-18	4.703E-18	1.950E-06	3.187E-17	9.598E-19	3.304E-18	3.304E-18
	outcome	≈	+	+	+	+	+	+	-	+	+
$F_{4}$	p-val	1	3.304E-18	3.304E-18	3.946E-14	1.491E-15	2.217E-07	5.370E-14	2.301E-06	3.304E-18	3.304E-18
	outcome	≈	+	+	+	+	+	+	+	+	+
$F_{5}$	p-val	5.184E-11	2.703E-13	8.868E-17	1.337E-15	8.868E-17	3.341E-04	1	3.393E-12	1	1
	outcome	-	+	+	+	+	+	≈	+	≈	≈
$F_{6}$	p-val	3.942E-18	3.304E-18	3.304E-18	3.304E-18	3.304E-18	2.534E-17	3.304E-18	1.091E-18	3.304E-18	3.304E-18
	outcome	+	+	+	+	+	+	+	-	+	+
$F_{7}$	p-val	1	3.304E-18	3.304E-18	3.304E-18	3.304E-18	1	6.227E-16	8.966E-20	3.304E-18	3.304E-18
	outcome	≈	+	+	+	-	≈	+	-	+	+
$F_{8}$	p-val	1.742E-11	3.304E-18	6.315E-17	1.194E-14	3.005E-15	1	2.912E-02	3.304E-18	4.181E-18	3.304E-18
	outcome	+	+	+	+	+	≈	+	+	+	+
$F_{9}$	p-val	1.009E-04	8.441E-18	8.498E-14	2.093E-11	4.662E-13	1	1	3.304E-18	3.304E-18	3.304E-18
	outcome	+	+	+	+	+	≈	≈	+	+	+
$F_{10}$	p-val	3.187E-17	3.304E-18	8.919E-08	4.006E-17	6.227E-16	2.217E-07	2.848E-15	3.304E-18	2.699E-15	3.304E-18
	outcome	+	+	+	+	+	+	+	+	+	+
$F_{11}$	p-val	7.659E-09	2.539E-10	1.150E-11	2.401E-11	2.003E-13	1.098E-11	1	2.382E-07	2.468E-07	6.741E-04
	outcome	-	+	-	-	+	-	≈	+	+	+
$F_{12}$	p-val	1.136E-15	3.160E-09	5.652E-14	4.241E-17	2.610E-14	3.151E-10	1	1.634E-14	1.363E-04	4.407E-06
	outcome	-	+	+	+	+	-	≈	+	+	+
$F_{13}$	p-val	5.126E-15	4.181E-18	2.683E-17	2.433E-16	2.132E-17	2.424E-15	3.946E-14	7.753E-11	3.304E-18	3.304E-18
	outcome	+	+	+	+	+	+	+	+	+	+
$F_{14}$	p-val	8.202E-03	3.304E-18	3.304E-18	1	4.466E-02	3.782E-11	2.580E-04	6.717E-03	3.942E-18	3.304E-18
	outcome	-	+	+	≈	+	+	+	-	+	+
$F_{15}$	p-val	3.956E-11	3.304E-18	3.304E-18	9.483E-18	2.392E-17	9.595E-03	2.259E-17	7.085E-18	3.304E-18	3.304E-18
	outcome	+	+	+	+	+	+	+	+	+	+
$F_{16}$	p-val	1	3.236E-08	1	3.608E-04	3.018E-10	2.127E-03	1	6.321E-03	4.538E-02	5.291E-05
	outcome	≈	+	≈	-	+	+	≈	+	+	+
$F_{17}$	p-val	1.496E-08	6.683E-17	3.304E-18	4.181E-18	3.304E-18	1.196E-17	7.485E-17	3.504E-18	3.304E-18	3.304E-18
	outcome	-	-	+	+	+	+	+	+	+	+
$F_{18}$	p-val	5.137E-04	4.738E-03	2.279E-09	2.684E-09	1.197E-07	9.178E-15	1.080E-09	1.137E-08	3.304E-18	3.304E-18
	outcome	+	+	+	+	+	+	+	+	+	+
$F_{19}$	p-val	1	3.304E-18	3.304E-18	3.504E-18	4.703E-18	3.015E-11	7.341E-16	5.739E-10	3.304E-18	3.304E-18
	outcome	≈	+	+	+	+	+	+	+	+	+
$F_{20}$	p-val	1.719E-02	6.683E-18	3.304E-18	4.241E-17	3.304E-18	1.634E-14	1.911E-14	1.066E-04	3.304E-18	3.304E-18
	outcome	+	+	+	+	+	+	+	+	+	+
$F_{21}$	p-val	3.301E-11	2.580E-04	3.304E-18	6.260E-14	2.874E-16	9.406E-14	2.941E-12	4.133E-06	3.304E-18	3.304E-18
	outcome	-	-	+	+	+	+	-	+	+	+
$F_{22}$	p-val	1	1.775E-03	3.387E-02	1	9.457E-04	7.095E-06	4.766E-04	4.662E-13	1.445E-05	3.304E-18
	outcome	≈	+	+	≈	+	+	+	+	+	+
$F_{23}$	p-val	1	3.304E-18	3.304E-18	3.304E-18	3.304E-18	7.400E-10	3.304E-18	1.391E-20	3.304E-18	3.304E-18
	outcome	≈	+	+	+	+	+	+	+	+	+
$F_{24}$	p-val	2.558E-15	3.304E-18	3.717E-18	1.129E-17	9.927E-17	5.326E-17	7.963E-18	3.232E-03	3.304E-18	3.304E-18
	outcome	+	+	+	+	+	+	+	+	+	+
$F_{25}$	p-val	8.441E-18	3.304E-18	3.304E-18	5.945E-18	4.434E-18	2.297E-15	4.753E-17	7.073E-17	3.504E-18	3.304E-18
	outcome	+	+	+	+	+	+	+	+	+	+
$F_{26}$	p-val	1.905E-13	1.664E-11	8.381E-17	2.328E-10	6.782E-11	2.881E-11	3.743E-10	3.648E-07	2.674E-12	3.304E-18
	outcome	+	+	+	+	+	+	+	+	+	+
$F_{27}$	p-val	1	1.946E-16	1.129E-17	1.196E-17	5.326E-17	3.304E-18	6.304E-18	2.679E-19	3.748E-14	3.304E-18
	outcome	≈	+	+	+	+	+	+	-	+	+
$F_{28}$	p-val	3.717E-18	3.304E-18	3.304E-18	2.132E-17	5.607E-18	3.304E-18	9.383E-17	5.124E-15	3.304E-18	2.572E-08
	outcome	+	+	+	+	+	+	+	-	+	+
$F_{29}$	p-val	9.694E-06	1.005E-17	4.434E-18	2.683E-17	1.508E-17	3.304E-18	3.504E-18	3.717E-18	3.304E-18	3.304E-18
	outcome	+	+	+	+	+	+	+	+	+	+
$F_{30}$	p-val	2.378E-03	4.987E-18	3.304E-18	2.259E-17	6.304E-18	3.375E-17	5.607E-18	5.194E-12	3.304E-18	3.304E-18
	outcome	+	+	+	+	+	+	+	+	+	+
	total ’+’, symbol	14	27	28	26	29	24	24	23	29	29

**Table 6:** Wilcoxon Signed-Rank Test Results for CEC2017 Benchmark Problems (BP); p-value denoted as p-val

BP		ILPE	LPE	GPEA	GPEae	GPEed	NeGPEs	TOGPEAe	DE	ABC	GWO
$F_{1}$	p-val	1	3.304E-18	3.304E-18	3.304E-18	3.304E-18	3.304E-18	1	4.987E-18	3.304E-18	3.304E-18
	outcome	≈	+	+	+	+	+	≈	+	+	+
$F_{2}$	p-val	1.256E-04	3.304E-18	3.792E-16	3.304E-18	4.987E-18	3.304E-18	1.337E-15	3.304E-18	3.304E-18	3.304E-18
	outcome	+	+	+	+	+	+	+	+	+	+
$F_{3}$	p-val	2.496E-02	3.304E-18	3.304E-18	3.304E-18	3.304E-18	3.304E-18	2.703E-13	3.304E-18	3.304E-18	3.304E-18
	outcome	-	+	+	+	+	+	-	+	+	+
$F_{4}$	p-val	1	3.304E-18	3.304E-18	3.304E-18	1.050E-16	3.304E-18	1.855E-09	5.098E-08	3.304E-18	3.304E-18
	outcome	≈	+	+	+	+	+	+	+	+	+
$F_{5}$	p-val	2.789E-04	3.304E-18	1.551E-14	3.304E-18	2.106E-13	3.304E-18	1	3.304E-18	3.304E-18	3.304E-18
	outcome	+	+	+	+	+	+	≈	+	+	+
$F_{6}$	p-val	1.693E-17	3.304E-18	3.304E-18	3.304E-18	3.304E-18	3.304E-18	6.933E-14	3.304E-18	3.304E-18	3.304E-18
	outcome	+	+	+	+	+	+	+	-	+	+
$F_{7}$	p-val	1.602E-04	3.504E-18	6.012E-15	3.304E-18	2.259E-17	3.304E-18	7.360E-09	3.304E-18	3.304E-18	3.304E-18
	outcome	+	+	+	+	+	+	+	+	+	+
$F_{8}$	p-val	1	3.375E-17	2.006E-12	3.304E-18	1.180E-06	3.304E-18	1	3.304E-18	3.304E-18	3.304E-18
	outcome	≈	+	+	+	+	+	≈	+	+	+
$F_{9}$	p-val	4.476E-16	9.483E-18	1.065E-17	3.304E-18	1.005E-17	3.304E-18	4.002E-06	3.304E-18	3.304E-18	3.304E-18
	outcome	+	+	+	+	+	+	+	-	+	+
$F_{10}$	p-val	1	3.304E-18	1	3.304E-18	6.578E-16	3.304E-18	1	3.304E-18	1.733E-05	3.779E-07
	outcome	≈	+	≈	+	-	+	-	+	+	+
$F_{11}$	p-val	6.194E-03	3.717E-18	3.304E-18	3.304E-18	2.539E-10	3.304E-18	2.147E-04	4.369E-15	3.304E-18	3.304E-18
	outcome	+	+	+	+	+	+	+	+	+	+
$F_{12}$	p-val	1	3.304E-18	3.304E-18	3.304E-18	5.749E-07	3.304E-18	1.900E-03	3.304E-18	3.304E-18	3.304E-18
	outcome	≈	+	+	+	+	+	+	+	+	+
$F_{13}$	p-val	4.611E-02	7.511E-18	3.504E-18	3.304E-18	5.579E-16	3.304E-18	4.604E-14	6.683E-17	3.304E-18	3.304E-18
	outcome	+	+	+	+	+	+	+	+	+	+
$F_{14}$	p-val	1	1.120E-02	3.304E-18	3.304E-18	1.825E-06	3.304E-18	3.387E-02	1.180E-12	3.304E-18	3.304E-18
	outcome	≈	-	+	+	-	+	-	+	+	+
$F_{15}$	p-val	1	5.184E-11	3.942E-18	3.304E-18	3.428E-09	3.304E-18	1	1.787E-05	3.304E-18	3.304E-18
	outcome	≈	+	+	+	+	+	≈	+	+	+
$F_{16}$	p-val	1	1	1	3.304E-18	1	3.304E-18	1	1.794E-17	5.051E-10	6.304E-18
	outcome	≈	≈	≈	+	≈	+	≈	+	+	+
$F_{17}$	p-val	1	1.478E-03	1	3.304E-18	1.291E-04	3.304E-18	6.110E-04	9.595E-03	1.990E-07	3.304E-18
	outcome	≈	+	≈	+	+	+	+	+	+	+
$F_{18}$	p-val	1.986E-02	1.239E-12	3.304E-18	3.304E-18	1.814E-14	3.304E-18	4.954E-12	4.434E-18	3.304E-18	3.304E-18
	outcome	+	+	+	+	+	+	+	+	+	+
$F_{19}$	p-val	2.147E-04	1	4.241E-17	3.304E-18	6.275E-09	3.304E-18	2.703E-13	1.817E-08	3.304E-18	3.304E-18
	outcome	+	≈	+	+	+	+	+	+	+	+
$F_{20}$	p-val	1	2.208E-02	1	3.304E-18	7.253E-04	3.304E-18	1	1.231E-02	4.544E-03	8.947E-18
	outcome	≈	+	≈	+	+	+	≈	+	+	+
$F_{21}$	p-val	3.523E-03	7.963E-18	4.508E-12	3.304E-18	1.211E-13	3.304E-18	1	3.304E-18	1.185E-02	3.304E-18
	outcome	+	+	+	+	+	+	≈	+	-	+
$F_{22}$	p-val	1	3.304E-18	3.304E-18	3.304E-18	3.304E-18	3.304E-18	3.304E-18	3.304E-18	3.304E-18	3.304E-18
	outcome	≈	+	+	+	+	+	+	+	+	+
$F_{23}$	p-val	1.300E-12	3.304E-18	3.304E-18	3.304E-18	5.607E-18	3.304E-18	2.325E-03	3.304E-18	2.132E-17	3.304E-18
	outcome	+	+	+	+	+	+	+	+	+	+
$F_{24}$	p-val	2.558E-15	3.504E-18	3.304E-18	3.304E-18	4.753E-17	3.304E-18	4.199E-07	3.304E-18	1.754E-15	3.304E-18
	outcome	+	+	+	+	+	+	+	+	+	+
$F_{25}$	p-val	1.540E-02	3.304E-18	3.304E-18	3.304E-18	5.637E-17	3.304E-18	5.962E-13	1.076E-15	3.304E-18	3.304E-18
	outcome	+	+	+	+	+	+	+	+	+	+
$F_{26}$	p-val	4.257E-02	1.900E-17	2.699E-15	3.304E-18	6.315E-17	3.304E-18	8.875E-03	3.304E-18	1	3.304E-18
	outcome	-	+	+	+	+	+	+	+	≈	+
$F_{27}$	p-val	1.347E-03	1.041E-13	3.304E-18	3.304E-18	5.184E-11	3.304E-18	2.453E-02	1.439E-08	1.851E-15	3.304E-18
	outcome	+	+	+	+	+	+	+	+	+	+
$F_{28}$	p-val	1	3.304E-18	3.304E-18	3.304E-18	2.572E-16	3.304E-18	1.855E-09	3.290E-10	3.304E-18	3.304E-18
	outcome	≈	+	+	+	+	+	+	+	+	+
$F_{29}$	p-val	1	1.858E-03	1.150E-11	3.304E-18	2.777E-08	3.304E-18	6.741E-04	6.683E-17	5.288E-18	4.703E-18
	outcome	≈	+	+	+	+	+	+	+	+	+
$F_{30}$	p-val	1	5.288E-18	3.304E-18	3.304E-18	7.795E-06	3.304E-18	1.066E-04	3.304E-18	3.304E-18	3.304E-18
	outcome	≈	+	+	+	+	+	+	+	+	+
	total ’+’, symbol	14	27	26	30	27	30	20	28	28	30

**Fig. 3:**
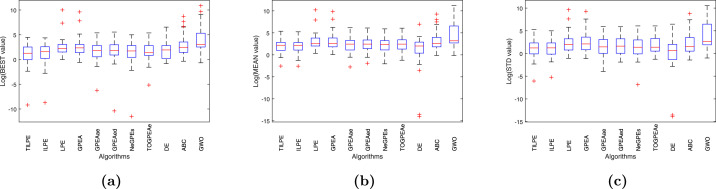
Box plots for CEC2014 benchmark functions; (a) for BEST, (b) for MEAN, (c) for STD

**Fig. 4:**
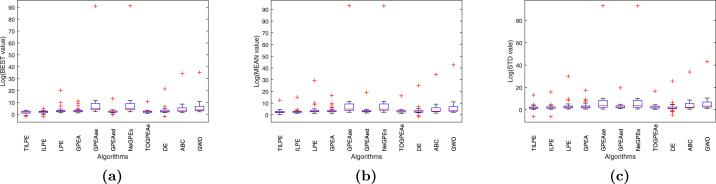
Box plots for CEC2017 benchmark functions;(a) for BEST, (b) for MEAN, (c) for STD

To conduct further analysis, we present the boxplots in Figs. 3 and 4, displaying the results of various metaheuristic algorithms calculated based on the BEST, MEAN and STD results. Figs. 3 and 4 demonstrate that the TILPE algorithm exhibits the minimum median value and the most compact box, indicating the stability of the algorithm’s results. Conversely, the other considered algorithms have broader boxes, indicating that their results are more spread from the median.

## Conclusion

The ILPE is a new, competitive evolutionary algorithm with a strong exploration capacity with fewer parameters. To exploit the feasible region more efficiently, a novel metaheuristic algorithm, namely TILPE, is presented in this paper. It incorporates the topological opposition-based learning before the selection operator of ILPE to accelerate the search process in order to find near-optimal solutions. By doing extensive experiments and analysis over CEC2014 and CEC2017 benchmark functions, the proposed algorithm proved its efficacy compared to ILPE, LPE, GPEA, GPEAae, GPEAed, NeGPEs, TOGPEAe, DE, ABC, and GWO algorithms.

## Ethical statements

None of the authors conducted studies involving human participants or animals in the preparation of this article.

## Funding

Author A. M. Mohiuddin has received research grant from South Asian University, India.

## Declaration of Competing Interest

The authors state that they do not have any competing financial interests or personal relationships that could have influenced the work presented in this paper.

## Data Availability

No data was used for the research described in the article.
